# RNA-Seq of three free-living flatworm species suggests rapid evolution of reproduction-related genes

**DOI:** 10.1186/s12864-020-06862-x

**Published:** 2020-07-06

**Authors:** Jeremias N. Brand, R. Axel W. Wiberg, Robert Pjeta, Philip Bertemes, Christian Beisel, Peter Ladurner, Lukas Schärer

**Affiliations:** 1 Department of Environmental Sciences, Zoological Institute, University of Basel, Vesalgasse 1, 4051 Basel, Switzerland; 2 Institute of Zoology and Center of Molecular Biosciences Innsbruck, University of Innsbruck, Innsbruck, Austria; 3 Department of Biosystems Science and Engineering, ETH Zürich, Basel, Switzerland

**Keywords:** Platyhelminthes, Orthologs, Rate of evolution, Regeneration, Differential expression, RNA-Seq

## Abstract

**Background:**

The genus *Macrostomum* consists of small free-living flatworms and contains *Macrostomum lignano*, which has been used in investigations of ageing, stem cell biology, bioadhesion, karyology, and sexual selection in hermaphrodites. Two types of mating behaviour occur within this genus. Some species, including *M. lignano*, mate via reciprocal copulation, where, in a single mating, both partners insert their male copulatory organ into the female storage organ and simultaneously donate and receive sperm. Other species mate via hypodermic insemination, where worms use a needle-like copulatory organ to inject sperm into the tissue of the partner. These contrasting mating behaviours are associated with striking differences in sperm and copulatory organ morphology. Here we expand the genomic resources within the genus to representatives of both behaviour types and investigate whether genes vary in their rate of evolution depending on their putative function.

**Results:**

We present de novo assembled transcriptomes of three *Macrostomum* species, namely *M. hystrix*, a close relative of *M. lignano* that mates via hypodermic insemination, *M. spirale*, a more distantly related species that mates via reciprocal copulation, and finally *M. pusillum*, which represents a clade that is only distantly related to the other three species and also mates via hypodermic insemination. We infer 23,764 sets of homologous genes and annotate them using experimental evidence from *M. lignano*. Across the genus, we identify 521 gene families with conserved patterns of differential expression between juvenile vs. adult worms and 185 gene families with a putative expression in the testes that are restricted to the two reciprocally mating species. Further, we show that homologs of putative reproduction-related genes have a higher protein divergence across the four species than genes lacking such annotations and that they are more difficult to identify across the four species, indicating that these genes evolve more rapidly, while genes involved in neoblast function are more conserved.

**Conclusions:**

This study improves the genus *Macrostomum* as a model system, by providing resources for the targeted investigation of gene function in a broad range of species. And we, for the first time, show that reproduction-related genes evolve at an accelerated rate in flatworms.

## Background

The genus *Macrostomum* (Platyhelminthes, Macrostomorpha) consists of small free-living flatworms and contains the model organism *Macrostomum lignano,* which has been used in numerous studies investigating a broad range of topics, ranging from sexual selection in hermaphrodites [1–3], ageing [4, 5] and stem cell biology [6], to bioadhesion [7–9] and karyology [10]. To enable this research many state-of-the-art tools have been established, such as an annotated genome and transcriptome [11, 12], efficient transgenesis [12], in situ hybridisation (ISH) [7, 13], and gene knock-down through RNA interference (RNAi) [3, 14]. The wealth and breadth of research on *M. lignano* make this species unique among the microturbellarians, for which research is generally restricted to taxonomic and morphological investigations.

**Fig. 1:**
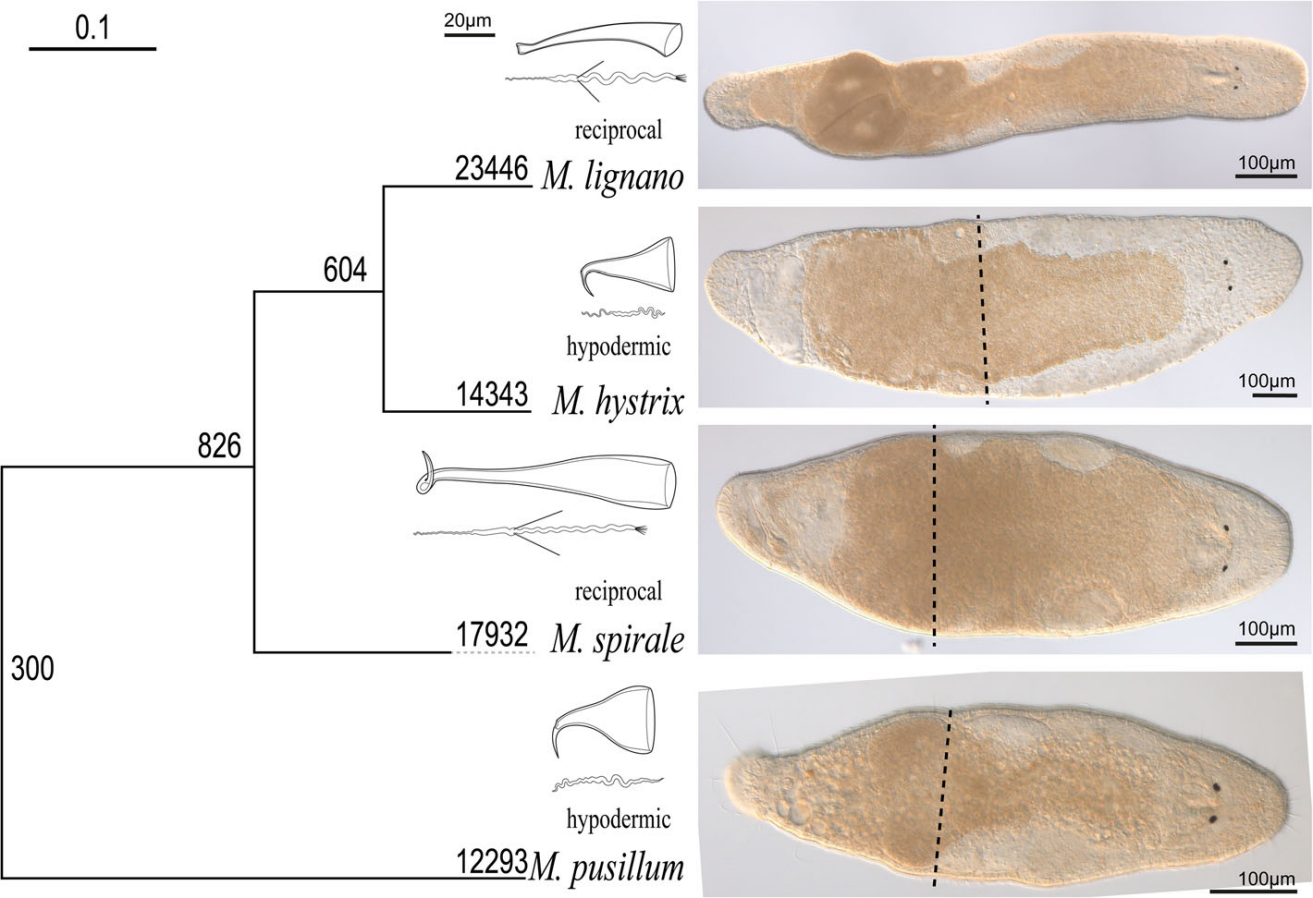
Details of the phylogenetic relationships and the morphology of the species in this study. Phylogeny of the four species (left) next to line drawings of the male copulatory organs (stylets) and sperm, and light microscopic images of lightly squeezed live worms. The type of mating (reciprocal/hypodermic) is indicated above the species name. The phylogeny (see also Results) is rooted at the branch leading to *M. pusillum* since this represents the deepest split in the genus (see [16]). The grouping of *M. lignano* with *M. hystrix* has maximal support (in both the ultrafast bootstrap as well as the Shimodaira–Hasegawa–like approximate likelihood ratio test), which suggests independent origins of the hypodermic mating syndrome in *M. hystrix* and *M. pusillum*. The scale bar represents substitutions per site, and the numbers next to the nodes give the number of gene duplications that occurred according to the OrthoFinder analysis (see also Methods; the amino acid alignment, the inferred phylogeny, and the log file of the IQ-TREE analysis are provided in Additional file 1: “Amino acid alignment of one-to-one orthologs**”**; Additional file 2: “Maximum likelihood phylogeny” and Additional file 3: “IQ-TREE logfile”). The stippled lines on the light microscopic images show the intended cutting level for the regenerant treatment (see also Methods)

Given the success of using *M. lignano* as a model system*,* it is now desirable to produce genomic resources for more species within the genus to test if insights gained in *M. lignano* can be generalised. This is especially relevant since two contrasting types of mating behaviour occur within this genus [15]. Some species, including *M. lignano* (Fig. 1), show the reciprocal mating syndrome. They mate via reciprocal copulation, where, in a single mating, both partners insert their male copulatory organ (the stylet) into the female sperm storage organ (the antrum), and simultaneously donate and receive sperm [15]. In addition, these reciprocally mating species possess stiff lateral bristles on their sperm, which are thought to be a male persistence trait to prevent the removal of received sperm [17]. Sperm removal likely occurs since, after copulation, worms of these species are frequently observed to place their pharynx over their female genital opening and then appear to be sucking, most likely removing seminal fluids and/or sperm from the antrum [18]. The sperm bristles could thus anchor the sperm in the epithelium of the antrum during this post-copulatory suck behaviour [17]. Other species within the genus, such as *M. hystrix*, show the hypodermic mating syndrome (Fig. 1). They mate via hypodermic insemination, where worms use a needle-like stylet to inject sperm into the tissue of the partner and the sperm then move through the tissue to the site of fertilisation [15, 19, 20]. Sperm of hypodermically mating species lack bristles entirely [15]. As a consequence of these contrasting mating behaviours there likely are differences in the function of reproduction-related genes between reciprocally and hypodermically mating species. Genomic resources for species with contrasting mating syndromes could, therefore, be used to identify these genes and investigate their function.

A range of empirical gene annotations derived from RNA-Seq experiments in *M. lignano* are available, with candidate gene sets that are differentially expressed (DE) between body regions [21], stages of tissue regeneration [22], social environments [23], animals of different ages [5], and between somatic cells and somatic stem cells (called neoblasts in flatworms) [6]. Identifying the homologs of genes with such empirical annotations in other *Macrostomum* species will allow us to investigate their function and rate of evolution in a broader phylogenetic context. For example, it can be assessed whether genes identified as being involved in neoblast function are conserved, and this may identify genes that are particularly important in flatworm regeneration.

Moreover, insights into the biology of these species can be gained by identifying rapidly evolving genes, since there is evidence that in a range of organismal groups reproduction-related genes evolve faster than genes serving other functions (reviewed in [24, 25]). Among the fastest-evolving genes are those encoding for proteins directly involved in molecular interaction with the mating partner, such as pheromone receptors (e.g. [26]), seminal fluid proteins (e.g. [27]), and proteins involved in gamete recognition and fusion (e.g. [28]). Groups of genes with biased expression in reproduction-related tissues, such as the testis and ovary, can also show elevated rates of evolution. Evidence for this comes both from sequence based analysis of the rate of divergence and the increased difficulty of detecting homologs of reproduction-related genes [29, 30].

Here we present transcriptomes and differential expression (DE) datasets of three *Macrostomum* species (Fig. 1; Additional file 1: “Amino acid alignment of one-to-one orthologs**”**; Additional file 2: “Maximum likelihood phylogeny” and Additional file 3: “IQ-TREE logfile”), namely i) *M. hystrix*, a close relative of *M. lignano* that mates via hypodermic insemination, ii) *M. spirale*, a somewhat more distantly related species that, like *M. lignano*, mates via reciprocal copulation, and finally iii) *M. pusillum*, which represents a clade that is deeply split from the other three species and which also mates via hypodermic insemination (see also [15, 16] for the broader phylogenetic context). All three species are routinely kept in the laboratory and studies have been published using cultures of *M. hystrix* [10, 19, 20, 31], *M. pusillum* [32], and *M. spirale* [10]. Since the comparison to *M. pusillum* represents one of the largest genetic distances within the genus, it is an ideal choice to identify genes that are either conserved or evolve rapidly. The inclusion of two species with hypodermic insemination furthermore allows candidate selection for genes involved in determining differences in sperm morphology.

**Fig. 2:**
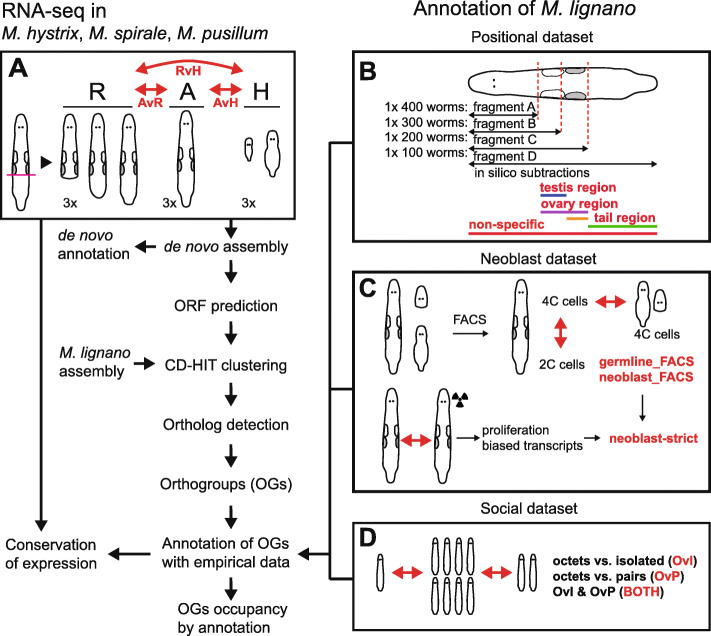
Flowchart of the analysis steps in the manuscript. The red double arrows indicate DE analyses and red labels the resulting DE annotations. **a** Details of the experiment conducted for this study (yielding three DE annotations: AvH, AvR, and RvH). **b** Details on the positional dataset of Arbore et al. (2015). The stippled red lines on the schematic drawing of the worm indicate the levels at which worms were amputated to produce the four fragments indicated below. These fragments were then used to identify genes that were DE in the body regions shown in colour (yielding four DE annotations: non-specific, testis region, ovary region, and tail region). **c** Details on the dataset of Grudniewska et al. (2016). The top row shows the identification of candidates using FACS and the bottom row the approach using irradiation to remove proliferating cells, permitting the annotation of transcripts with germline- and neoblast-biased expression (yielding three DE annotations: germline_FACS, neoblast_FACS, and neoblast-strict). **d** Details of the social dataset of Ramm et al. (2019). Comparisons between worms grown in different social group sizes permit identifying socially-sensitive transcripts (yielding three DE annotations: OvI, OvP, and BOTH)

In all three species, we produced RNA-Seq libraries for adults (A), hatchlings (H), and regenerants (R), in order to capture the expression of as many genes as possible and to allow for DE analyses between these biological conditions (Fig. 2a, red labels). Since hatchlings lack sexual organs, genes with higher expression in adults compared to hatchlings can serve as candidate genes that are specific for those organs. Conversely, genes with higher expression in hatchlings are candidates for genes regulating early development. Finally comparing gene expression in adults vs. regenerants can identify regeneration-related candidate genes involved in the development of structures that are not actively forming in the adult steady state, such as the male genitalia (as demonstrated in [22]). Besides conducting the described DE analysis, we also determined groups of homologous genes (called orthogroups [OGs] throughout the text) between the three species presented here and *M. lignano* (Fig. 2). This allowed us to transfer the empirical annotations from three RNA-Seq experiments performed in *M. lignano* (Fig. 2b-d, red labels) to these inferred OGs and investigate whether OGs with particular annotations show signs of conservation or rapid evolution in patterns of protein sequence divergence and/or gene presence/absence.

## Results

### Transcriptome assembly and quality

We used > 300 million paired-end reads per species—derived from adults (A), hatchlings (H), and regenerants (R)—to assemble the transcriptomes of *M. hystrix*, *M. spirale*, and *M. pusillum* (Table 1). All three transcriptomes were fairly complete in gene content when assessed using BUSCO, with more than 92.5% of all 978 core metazoan genes found either complete or as fragments in all species (Table 1). Moreover, the assemblies were a good representation of the reads used to infer them, with > 87 and > 46% of the reads mapping back to the raw and the (CD-HIT) reduced assembly, respectively (Table 2). TransRate scores were between 0.28 and 0.29 (Table 1), placing them above average when compared to 155 publicly available transcriptomes evaluated in [33] (which ranged from 0 to 0.52, with an average of 0.22). The *M. spirale* transcriptome contained almost twice as many transcripts as the other two, but although *M. spirale* had the highest absolute number of functional annotations (Table 1), it had the lowest percentage of transcripts with annotations. The *M. spirale* assembly could thus contain more redundant sequences, contain more poorly assembled contigs due to increased heterozygosity or contain more non-coding transcripts than the others (see Discussion).

**Table 1:** Transcriptome assembly statistics per species. The initial number of reads used, the number of reads after Trimmomatic processing, the number of initially assembled transcripts, the empirical mean insert size of the RNA-Seq libraries, the number of distinct 21-mers, the number of transcripts removed by CroCo, and the final number of transcripts, as well as the mean transcript length and number of bases in the final assemblies are shown. The BUSCO score is given as the percentage of complete (C) genes—divided into present as single copies (S) or duplicates (D)—and fragmented (F) genes of the 978 metazoa gene set. The next three rows detail the TransRate score, the number of transcripts remaining after TransDecoder translation and CD-HIT clustering, and the number of transcripts considered in the DE analysis. Below this a summary of the results from the Trinotate annotations giving the number of transcripts (and the corresponding percentage of the whole transcriptome in brackets) with a given annotation: ORF, contains a predicted open reading frame; BLASTX, the predicted ORF and/or the entire transcript produced a hit in the protein database; Pfam, a protein family domain was found; SignalP, a signal peptide was detected; TMHMM, a transmembrane helix is predicted

Assembly statistics	*M. hystrix*	*M. spirale*	*M. pusillum*
Initial reads	160,231,340	173,766,431	157,755,458
Reads post trimming	148,699,208	160,248,517	147,615,465
Mean insert size	146	143	145
Distinct 21-mers	160,907,099	235,628,648	194,772,389
Assembled transcripts	169,758	296,658	177,453
Removed transcripts	217	156	274
Final transcripts	169,541	296,502	177,179
Mean transcript length	1094	764	756
Number of bases	185,792,353	226,578,146	134,085,334
BUSCO score (Metazoa gene set)	C: 90.1 S: 49.3 D: 40.8 F: 3.4	C: 87.8 S: 37.3 D: 50.5 F: 4.7	C: 89.2 S: 55.8 D: 33.4 F:4.1
TransRate score	0.28	0.29	0.28
CD-HIT transcripts	53,132	74,135	53,416
DESeq2 transcripts	43,126	66,139	41,418
**Annotation**			
ORF	59,889 (35.3)	70,808 (23.9)	49,456 (27.9)
BLASTX	47,837 (28.2)	50,033 (16.9)	42,940 (24.2)
Pfam	42,330 (25.0)	43,840 (14.8)	34,726 (19.6)
SignalP	6486 (3.8)	6601 (2.2)	5380 (3.0)
TMHMM	15,399 (9.1)	16,322 (5.5)	14,537 (8.2)

**Table 2:** Read mapping statistics. The average percentage of reads per species and condition, which could be mapped back to the raw or reduced transcriptome assemblies, respectively

Species	Condition	Mapped to raw assembly (%)	Mapped to reduced assembly (%)
*M. hystrix*	Adult (A)	93.4	68.9
	Hatchling (H)	92.9	68.0
	Regenerant (R)	94.1	64.1
*M. spirale*	Adult (A)	88.1	48.3
	Hatchling (H)	87.0	51.1
	Regenerant (R)	88.7	46.0
*M. pusillum*	Adult (A)	90.8	74.1
	Hatchling (H)	89.0	73.0
	Regenerant (R)	91.7	74.1

### Orthology detection

We used OrthoFinder to infer 23,764 OGs, with 11,331 of those OGs containing sequences from all four species, and 1190 containing all species except for *M. lignano* (see Additional file 4: Table S1 for all inferred OGs). OGs were generally large with only 1263 single-copy orthologs identified between all four species (these orthologs were used for the species tree inference depicted in Fig. 1, see also below). OrthoFinder provides a summary of the number of gene duplications that occurred on each node of the species tree (Fig. 1), and this analysis indicated that most of the gene duplications occurred on the terminal branches, with the highest number occurring in *M. lignano*.

### DE analysis

**Fig. 3:**
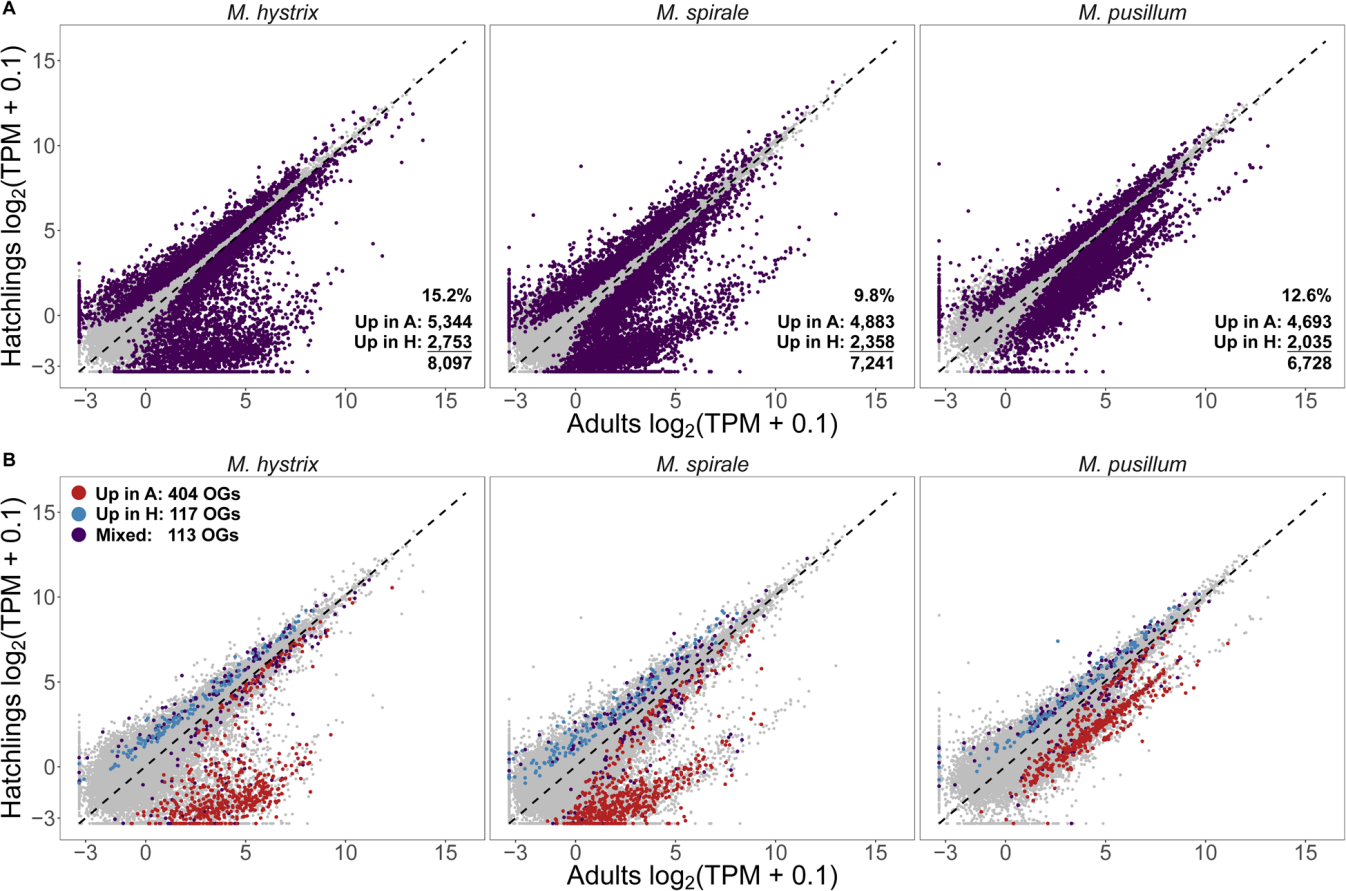
Results of differential expression (DE) analysis between adults and hatchlings. **a** Results of DE analysis comparing the expression in adults (shown on the x-axis) against expression in hatchlings (shown on the y-axis). Highlighted are transcripts that are significantly DE after adjusting for multiple testing (adjusted *p*-value < 0.05). The numbers at the bottom right of each panel refer to the total number of DE transcripts, and the percentage of DE transcripts out of all transcripts. **b** The same plots, but highlighting only transcripts from OGs that have representatives in all three species (but not necessarily a transcript from *M. lignano*) and in each species at least one transcript that is DE. Transcripts in red are significantly upregulated in adults, transcripts in blue are significantly upregulated in hatchlings, and transcripts in purple show an inconsistent signal within the OG

When comparing expression of adults vs. hatchlings (AvH), similar numbers of transcripts were DE in all three species, with about twice as many transcripts with higher expression in adults compared to hatchlings (Fig. 3a, see also Additional file 5: Table S2 for the DE results of the AvH comparison, and Additional file 6: Table S3 and Additional file 7: Table S4 for the AvR and RvH contrasts). *M. pusillum* showed slightly lower numbers of DE genes and a DE distribution that deviated from that of the other two species. Specifically, the distributions of DE genes in both *M. hystrix* and *M. spirale* shows a cloud of off-diagonal points, representing transcripts with high expression in adults, but low expression in hatchlings. In *M. pusillum,* this cloud of adult-biased transcripts also exists, but it is shifted up on the y-axis because many of these transcripts also show substantial expression in hatchlings.

We identified a total of 634 OGs that had at least one transcript from every species DE in the AvH contrast (Fig. 3b). 404 of these showed higher expression in adults, 117 showed higher expression in hatchlings, and 113 did not have a consistent signal. Again, we observed differences between *M. pusillum* and the other two species. All but two of the transcripts in those with higher expression in adults also had expression in hatchlings, while in *M. hystrix* and *M. spirale* many transcripts had no expression in hatchlings (see points with red colour at the bottom of the y-axis in Fig. 3b). We explore possible reasons for these observations in the Discussion.

### Orthogroup annotation

18,938 OGs contained transcripts from *M. lignano* and could thus potentially carry over empirical annotations. Out of these, 6119 OGs could be annotated with information from the positional (2495 OGs), neoblast (1924 OGs), or social (3717 OGs) RNA-Seq datasets (see Additional file 8: Table S5 for the full annotations). In the positional dataset 173 OGs contain Mlig_37v3 transcripts with conflicting positional information (e.g. tail region and testis region). We categorised these as “positional_mix” and did not consider them further in the downstream analysis since they contain multiple small groups with non-intuitive annotations. Similarly, in the neoblast dataset, we categorised 20 OGs as neoblast_mix because they contained transcripts with the germline annotation (germline_FACS) and transcripts with one of the two neoblast annotations (neobast_FACS and neoblast-strict). Finally, in the social dataset, we categorised 10 OGs as social_mix because they contained transcripts with the octets vs. isolated annotation (OvI) annotation and transcripts with the octets vs. pairs (OvP) annotation, but no transcript annotated from both contrasts (BOTH). We also excluded both the neoblast_mix and the social_mix annotations from the downstream analysis.

**Fig. 4:**
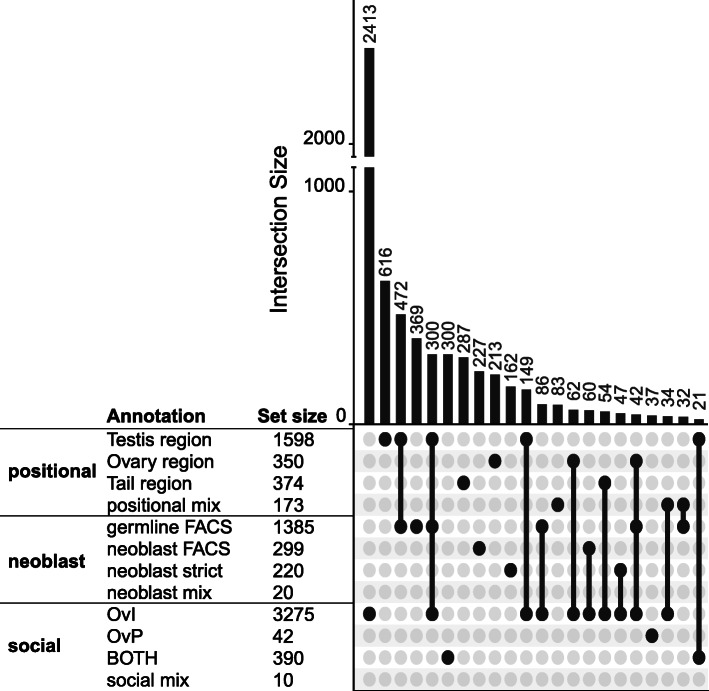
Upset plot of the intersection of orthogroup (OG) annotations. Annotations are from the positional (testis region, ovary region, tail region, and positional_mix), neoblast (germline_FACS, neoblast_ FACS, and neoblast-strict), and social datasets (OvI, OvP, and BOTH). The dots and lines on the bottom right show which intersection is represented by the bar plots above. The size of intersections is given above the bar plot. To the left of the intersection diagram, the absolute number of OGs per annotation is given. Note that only intersections with > 20 OGs are displayed here, but that the set sizes reflect the sums of all OGs (for a complete plot see Additional file 9: Fig. S1)

There was also overlap between the three RNA-Seq datasets, with several OGs being annotated from multiple sources. The most substantial overlap was between the germline_FACS and the testis region annotation, followed by the overlap between these two annotations and the octets vs. isolated (OvI) annotation (Fig. 4 and Additional file 9: Fig. S1). This overlap was expected since testis region transcripts likely contain mostly transcripts expressed in the testes. Since the neoblast annotation was independent from our reanalysis of the positional dataset, the considerable overlap it shows with the positional and social data supports that these annotations are indeed reflecting biological reality. However, this overlap also made them highly redundant, and we thus excluded the germline annotation from the downstream analysis, retaining only the neoblast annotations. Within the social dataset, most OGs were either annotated as OvI or as BOTH, while only 42 OGs carried the OvP annotation. We also excluded the OvP annotation due to small sample size, leaving us with seven DE annotations in total for the downstream analysis (testis region, ovary region, and tail region; neoblast_FACS and neoblast-strict; and OvI and BOTH; but see Additional file 10: Table S6 for a complete annotation of the Mlig_37v3 transcriptome).

The distribution of secretory signals, as estimated by SignalP, was not uniform across the different positional annotations (chi-squared = 18.0, df = 4, *p*-value = 0.001). The observed counts only differ substantially from the expected counts for the tail region OGs (54 observed vs. 32.9 expected, Table 3), indicating that OGs in the tail region are enriched in transcripts with a secretory signal.

**Table 3:** SignalP enrichment analysis. The number of complete OGs that contain transcripts with a SignalP hit, split by the positional annotation. The expected number of OGs with a SignalP is derived from the chi-square test

Annotation	OGs with annotation	OGs with SignalP	Expected SignalP
Testis region	728	130	128.5
Ovary region	181	37	31.9
Tail region	173	53	30.5
Positional_mix	84	16	14.8
No annotation	10,165	1764	1794.2

### Protein divergence and species composition of OGs differs by annotation

**Fig. 5:**
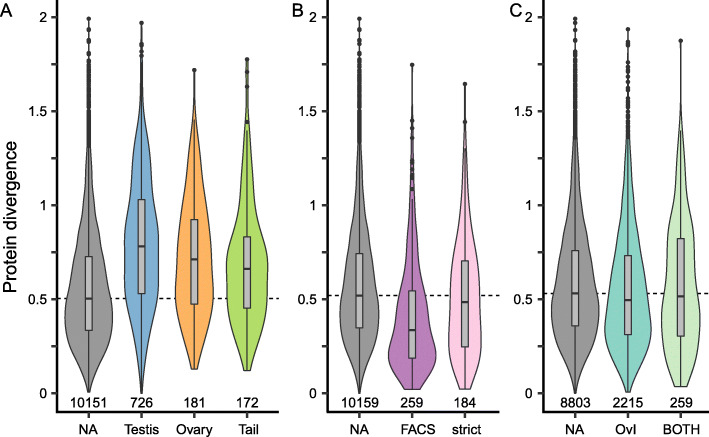
Violin (mirrored density) plots and boxplots (median, box shows the interquartile range and whiskers extend up to 1.5 times the interquartile range) of the distribution of average protein divergence of OGs with various annotations. Numbers above the x-axis give the number of OGs in each group. **a**: OGs with a positional annotation (excluding the positional_mix annotation). **b**: OGs with a neoblast annotation: neoblast_FACS (FACS) and neoblast-strict (strict) (excluding the germline_FACS and neoblast_mix annotations). **c**: OGs with a social annotation (excluding the OvP and social_mix annotations). The stippled lines represent the median values of the respective OGs with no annotation (NA) against which the OGs with an annotation were tested (see Results)

**Fig. 6:**
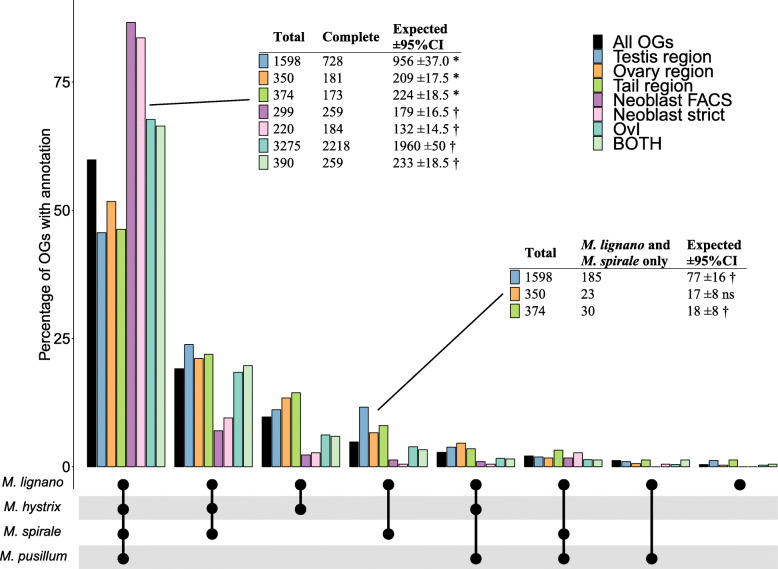
Species composition of Orthogroups (OG) with different annotations. The bar charts indicate the percentage of OGs with the species composition drawn below. The colours identify OGs with different annotations (see legend top right). The exact numbers and percentages for each annotation and species composition can be found in Additional file 11: Tab. S7. The two inset tables give the results of permutation tests to investigate if fewer or more than the expected number of OGs with a particular annotation contain all four species (i.e. are complete, table on the left) or contain only *M. lignano* and *M. spirale* (table on the right) (see Methods for details). In both tables, the first column gives the total number of OGs with the annotation and the second column gives the number of OGs that are complete (left table) or restricted to *M. lignano* and *M. spirale* (right table). The third column gives the expected number of OGs derived from 100,000 samples from all OGs and the corresponding 95% confidence interval. Symbols indicate if the observed value deviates from the expected value (* = significantly smaller than expected, † = significantly larger than expected, ns = not significantly larger or smaller). All significant *p*-values are < 0.01, all tests were two-tailed, and corrected for multiple comparisons

The majority (59.8%) of OGs with a transcript from *M. lignano* contained all four species and 19.1% contained all species except *M. pusillum*, while only a few (1.2%) were shared just between *M. lignano* and *M. pusillum* (Additional file 11: Table S7). The protein divergence of OGs containing all four species differed depending on their annotation, with higher divergence in OGs with a positional annotation (one-sample Wilcoxon: all *p* < 0.001, Fig. 5a) and lower divergence in OGs with the neoblast_FACS annotation (one-sample Wilcoxon: *p* < 0.001), but not the neoblast-strict annotation (one-sample Wilcoxon: *p* = 0.2, Fig. 5b) compared to OGs without an annotation from the respective sources. These patterns of divergence were also reflected in the species composition of OGs, with a smaller than expected percentage of OGs with a positional annotation containing all four species (Fig. 6), which is consistent with the more rapid evolution of these putative reproduction-related transcripts. Conversely, a substantially larger percentage of OGs with either of the neoblast annotations contained all four species (Fig. 6), suggesting that these genes are fairly conserved. Finally, while OGs annotated with the social dataset did not show a difference in protein divergence compared to OGs with no annotation (one-sample Wilcoxon: OvI: *p* = 0.34, BOTH: *p* = 0.34, Fig. 5 C) they contained a larger than expected percentage of OGs with all four species (Fig. 6). The difference between the expected and observed proportions was, however, quite small for the ‘BOTH’ annotation (Fig. 6), indicating a small effect size. Moreover, OGs annotated as testis or tail region contained a higher than expected percentage of OGs that were shared only between *M. lignano* and *M. spirale* (Fig. 6). Since both of these species mate through reciprocal copulation and have a characteristic sperm morphology with lateral bristles [15], these OGs are possible targets in the search for the genes underlying these traits. We explore these observations in more detail in the Discussion.

### OG validation using ISH

**Fig. 7:**
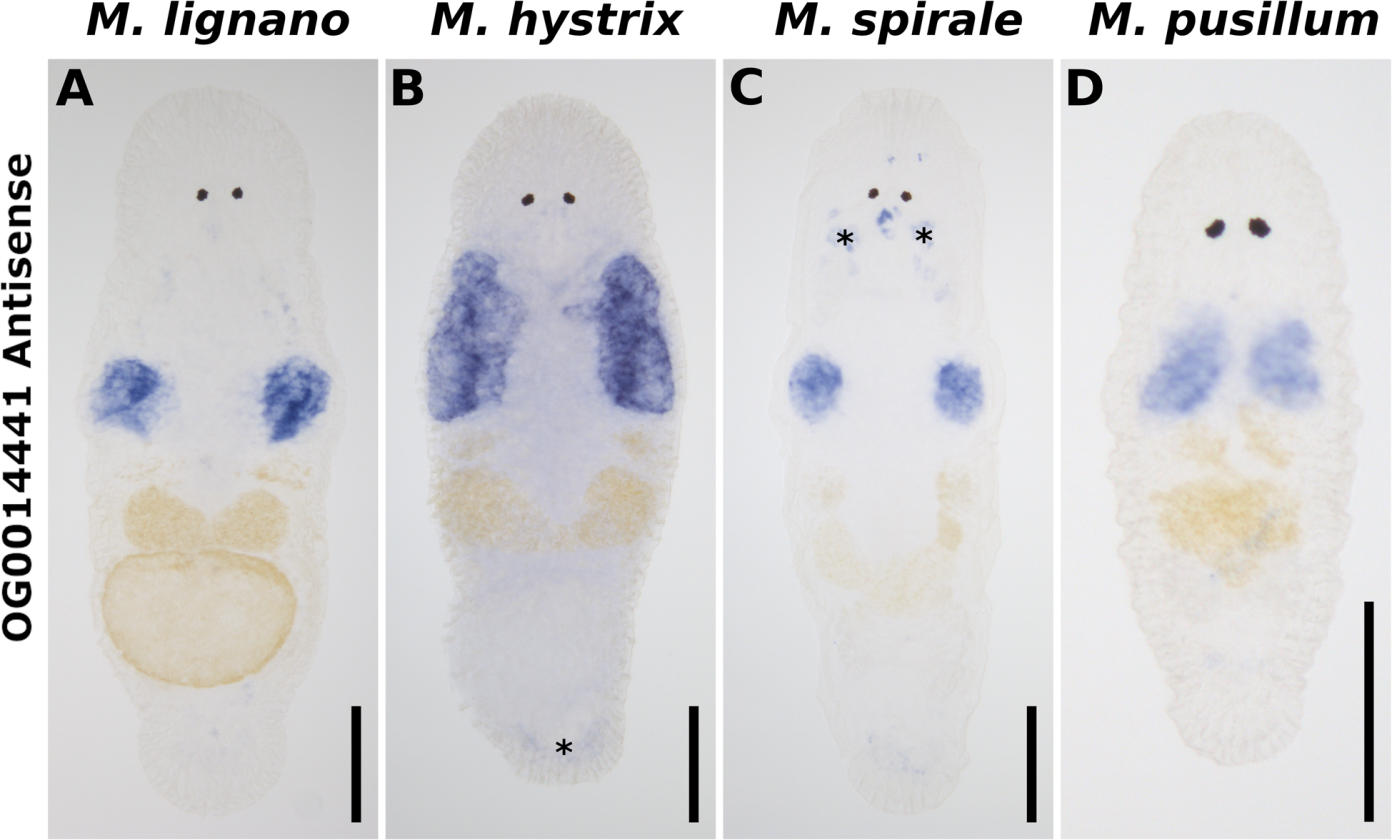
Whole-mount in situ hybridisations for OG0014441. Expression of the transcript affecting the sperm bristle phenotype and its orthologs in the testes of in *M. lignano* (**a**), *M. hystrix* (**b**), *M. spirale* (**c**), and *M. pusillum* (**d**). Unspecific staining (asterisks) can be seen in pharyngeal glands and in the tail region (staining of pharyngeal glands is also present in control experiments with the sense probe; see also Additional file 12: Fig. S2). Transcripts RNA815_7008 (*M. lignano)*, Machtx_20180703@G07456_i1 (*M. hystrix*), Macspi_20180703@G161928_i1 (*M. spirale*), and Macpus_20180703@G35224_i1 (*M. pusillum*) of orthogroup OG0014441 were used for in situ probe generation. Scale bars: 100 μm

As a case study to show the relevance of the OGs across all four studied species, we analysed the expression of a gene that affects the sperm bristle phenotype in *M. lignano* (RNA815_7008 in the MLRNA110815 transcriptome) [21]. This transcript is exclusively expressed in the testes in *M. lignano* [21], and we thus expect its orthologs to also be expressed in the testes of the other species. We designed probes for the orthologs in *M. hystrix*, *M. spirale*, and *M. pusillum* and performed ISH experiments to test this prediction. In addition, we also repeated the ISH experiments in *M. lignano*. We detected a highly specific signal in the testes in all four species (Fig. 7.; for sense control see Additional file 12: Fig. S2), which i) indicates that tissue specificity of this transcripts is conserved across the genus, and ii) demonstrates that our OGs can be used to identify orthologs and target them using molecular methods.

## Discussion

In the following section, we will first highlight some differences in the transcriptome assemblies and the DE results between the three species and their possible influence on our conclusions. Then we will focus on the differences in protein sequence divergence and species composition of OGs by annotation and discuss their implications. Note that we were only able to arrive at these results because we spent considerable effort on the reannotation of the *M. lignano* transcriptome. We discuss the majority of this work in Additional file 13: ‘Reannotation of Mlig_37v3 transcriptome’ (which also makes reference to Additional file 14: Table S8; Additional file 15: Table S9; and Additional file 16: Table S10) and we direct the reader to this document for a detailed explanation of all annotations.

### Transcriptome assembly and quality

By performing an analysis of the transcriptome assemblies, we found that the transcriptome of *M. spirale* contained almost twice as many transcripts than the other two transcriptomes. In spite of this, a similar number of annotations was generated in *M. spirale* as in the *M. hystrix* transcriptome, indicating that many of the additional transcripts in the *M. spirale* transcriptome represent either redundant sequences, poor assembly, or non-coding RNA. Poor assembly could be caused by a shorter library insert size, which would make it more difficult to span repetitive sequences. However, insert size of the *M. spirale* libraries was not substantially smaller than that of the other libraries. We think it is more likely that the *M. spirale* libraries contain more heterozygosity, potentially leading to erroneous assembly of alleles as separate contigs. The *M. hystrix* specimens we used originate from the highly inbred SR1 line, and *M. pusillum* can self-fertilise, showing no signs of inbreeding depression [32]. In contrast, the *M. spirale* specimens stem from an outbred culture and might, therefore, retain higher rates of heterozygosity. This is also supported by the 21-mer diversity of the input reads, with the highest number of distinct 21-mers found in *M. spirale* (Table 1). The additional complexity of the *M. spirale* assembly resulted in a substantially lower number of reads mapping to the reduced assemblies (Table 2), potentially diminishing our ability to detect DE in genes with low expression. However, since we are primarily interested in genes that show strong expression differences between adults vs. hatchlings, this should not adversely affect our conclusions.

### Orthology detection

The orthology detection indicated a high number of gene duplications, particularly with respect to the branches leading to each species. Duplications in *M. lignano* are expected, since it has recently been shown to be a hidden tetraploid, having undergone a whole-genome duplication [34, 35]. The ‘normal’ karyotype of *M. lignano* consists of three small and one large chromosome (2n = 8), with some specimens having additional copies of the large chromosome (and more rarely also other aneuploidies) [34]. *M. lignano*’s large chromosome represents a fusion of much of the genetic material found on the three smaller chromosomes [35], suggesting that the majority of the genes are present in duplicate. However, there is no evidence for such a duplication in *M. hystrix* or *M. spirale* (with both species being 2n = 6 with three small chromosomes, [10]), while the situation in *M. pusillum* is less clear (being 2n = 12 with six small chromosomes, Zadesenets et al. unpublished). It is unclear why we detect such high levels of gene duplication. One possibility is the presence of isoforms or alleles that cannot be clustered using the CD-HIT algorithm. The second highest number of duplications is present in *M. spirale*, which likely also is most heterozygous. To investigate this further, it would be necessary to produce genome assemblies of these species and to refine the gene models using genome-guided transcriptome assembly, as it was done for *M. lignano* [12, 36].

### DE analysis

The observed expression differences between adults and hatchlings are less pronounced in *M. pusillum* compared to the other two species, with fewer transcripts exclusively expressed in adults, and a general shift to higher expression in hatchlings for transcripts detected as DE in adults. *M. pusillum* has a shorter generation time than the other two species [32], and this could have presented challenges when we collected the hatchlings, leading us to collect worms that had already become subadults and started to express some reproduction-specific transcripts, albeit likely at a relatively low level. Since our hatchling samples were intended to contain various life-stages up to early juveniles, it would be interesting to generate additional RNA-Seq data of even younger *M. pusillum*. If we indeed collected some subadults in the *M. pusillum* hatchling pools, then we would expect the DE patterns between these pools and adults to be more similar compared to the patterns observed in *M. hystrix* and *M. spirale*.

### Protein divergence and species composition of OGs differs by annotation

OGs annotated as specific for the testis, ovary, or tail region had higher average protein divergence than OGs with no annotation, suggesting a faster rate of evolution in these reproduction-related genes (Fig. 5). Additionally, OGs annotated with these reproduction-related annotations generally contained fewer species than a random subset of OGs (Fig. 6). While the patterns in species composition could also be produced by processes like gene duplication, gene gain/loss, or introgression, our findings are consistent with previous research showing that reproduction-related genes evolve more rapidly [24, 25] and that a major cause of homology detection failure across species is sequence evolution to the point where homology detection algorithms fail [37, 38]. More thorough testing of this hypothesis will require fitting explicit models of sequence evolution to the data. An important caveat is that the positional annotation does not identify the testis, ovary, and prostate directly, but merely the regions containing these organs. It could, therefore, be that we also include non-reproduction related genes in these analyses. This is undoubtedly the case for the tail region since the tail contains organs that are not present in other parts of the body, such as the adhesive organs as well as the shell and cement glands used for eggshell formation and adhesion of the eggs to the substrate [7, 9, 39]. But this can also be the case for the testis and ovary region since there appears to be a specialised gut epithelium in this area [21]. However, we think it is a valid assumption that reproduction-related genes represent a large proportion of transcripts with a positional annotation. The best evidence for this comes from ISH experiments, which showed that the majority of the tested transcripts in the ovary and testis region are indeed expressed in the gonads [21]. This is further supported by our ISH experiment showing that the orthologs of a gene expressed exclusively in the testis in *M. lignano* are localised in the same way in *M. hystrix*, *M. spirale*, and *M. pusillum.* Furthermore, detailed screening of the tail region transcripts identified a large proportion as expressed in the prostate gland cells [40]. We also screened our gene annotations of OGs with a positional annotation for the presence of a signal peptide and found that the tail region is enriched in this way. This further supports that we are capturing reproduction-related genes since genes expressed in the prostate have also been shown to be enriched with secretion signals [40]. Finally, including non-reproduction related transcripts in the positional annotations should dilute the signal of increased sequence divergence and thus make our test more conservative, which further supports our finding.

Interestingly, we found an increased proportion of OGs with a testis or tail region annotation that only contained *M. lignano* and *M. spirale*. While the number of the latter is low and thus needs to be interpreted with some caution, the number of the former is quite large, so that this finding can be considered well supported. Since both of these species mate through reciprocal copulation and have a characteristic sperm morphology with lateral bristles [15], these OGs are possible targets in the search for the genes underlying these traits.

In contrast to the OGs with positional annotations, we showed that OGs with one of the neoblast-specific annotations (neoblast_FACS) had lower average protein divergence than the corresponding OGs with no annotation (Fig. 5) and that OGs annotated with both neoblast annotations were more likely to contain all four species (Fig. 6). This suggests that these transcripts are conserved across the genus, as one might expect for stem cell genes since they perform essential functions in homeostasis and regeneration. However, we were not able to place all of the clusters that were annotated with the neoblast dataset in *M. lignano* into an OG (Additional file 13: see Table A2 therein). This is despite the fact that many of these clusters appear to have a human homolog according to a BLAST search [6]. The failure to identify these transcripts in other *Macrostomum* species could be due to poor assembly of the homologous transcripts, but more likely is due to the challenges inherent in orthology detection. Orthology detection methods have to balance the trade-off between precision (the correct identification of orthologous relationships) with recall (the total number of genes grouped into OGs) [41, 42]. To avoid spurious grouping of transcripts, these methods, therefore, discard a substantial number of them, leading to a reduction in recall. We do not contend that transcripts in *M. lignano* that were not placed into an OG have no homologs in the other species. Rather, their lack of placement is likely a consequence of the decisions made within the OrthoFinder algorithm, which has been shown to have similar performance compared to other available orthology detection methods (see benchmarks on orthology.benchmarkservice.org). Furthermore, we consider orthology detection methods like OrthoFinder that rely on explicit modelling of gene trees preferable to approaches relying on similarity only. Approaches using gene trees do more explicitly model evolutionary history and should be more accurate in the presence of gene duplications (as present in *M. lignano* [34]), gene loss, and incomplete assemblies [43, 44].

Finally, we could show that OGs annotated by the social dataset have higher species occupancy, indicating that they are more conserved compared to a random subset of OGs, while they did not show a difference in protein divergence. This is a somewhat counter-intuitive finding since these annotations are reproduction-related, showing the change in expression to the availability of a mate (OvI), the intensity of sperm competition (OvP), or to both (BOTH). Therefore, we would expect a large overlap between social annotations and the germline_FACS as well as the positional annotations. In the original publication, Ramm et al. [23] determined that a large proportion of transcripts with a positional annotation are DE in response to mating (see Fig. 4 in [23]), which is also what we find in our reanalysis of their data (Additional file 13: see Fig. A3 and Table A3 therein). Indeed, some OGs annotated as OvI show overlap with the testis region annotation and/or the germline_FACS annotation, but the majority have no overlap with other annotations (Fig. 4). Additionally, there is overlap between the OvI annotation and the neoblast dataset (Fig. 4). This overlap offers a possible explanation for the conservation of socially sensitive genes. In response to mating, multiple physiological changes occur, resulting in a general increase in metabolic activity, which could lead to a higher expression of transcripts involved in general maintenance of cellular processes. Alternatively, these transcripts could be regulating more general sensory or neurological traits used for the sensing of conspecifics. Transcripts involved in such fundamental processes are expected to be conserved, which would fit with our observation. OGs annotated with the social dataset are thus likely a heterogeneous population consisting both of reproduction-related genes and general metabolic genes.

## Conclusions

The three high-quality transcriptomes and the accompanying DE data, in combination with an annotated set of OGs, will facilitate candidate selection for further investigations of gene function across the genus *Macrostomum*. Particularly interesting in this respect are the OGs with consistent DE across all species, as well as OGs that only contain *M. lignano* and *M. spirale*. These OGs are possible targets to identify reproduction-related genes and should be investigated using molecular techniques such as ISH and RNAi.

We show that reproduction-related genes evolve rapidly within the genus *Macrostomum*. To our knowledge, this is the first evidence for the rapid evolution of such genes in flatworms and the first evidence for this phenomenon in hermaphroditic animals, since previous research has focused almost exclusively on separate sexed organisms. Future investigations should expand taxon sampling to validate this finding, and expand analyses beyond documenting differences in the species composition of OGs and simple protein distances among species. Future studies could use sequence-based approaches such as the estimation of the rate of non-synonymous to synonymous substitution to identify particular genes that evolve rapidly [45].

Finally, our annotations are derived from *M. lignano* and are thus taxonomically biased. We suggest future work to replicate experiments conducted with *M. lignano* in other species, which would allow a more independent and balanced annotation of the OGs. This then would allow validation of the OG annotations as well as permit the annotation of genes that evolve too rapidly to be currently assigned to an annotated OG.

## Methods

### Animal cultures

The specimens of *Macrostomum hystrix* Ørsted 1843 sensu Luther 1905 used in this study originate from an inbred line derived from an outbred culture initially collected in May 2010 from a brackish canal in the San Rossore Regional Park, near Pisa, Italy (N43.6848, E10.2838; note that the name *M. hystrix* is taxonomically problematic, as outlined in [15]). After the discovery that *M. hystrix* can self-fertilise [19], the inbred SR1 line was generated by enforcing selfing for eight generations, followed by several generations of sib-sib breeding (predicted inbreeding coefficient, F = 0.998, [31]). The specimens of *Macrostomum spirale* Ax 1956 derive from a long-term outbred laboratory culture initially collected in November 2004 from the very mouth of the Étang de Biguglia in Corsica, France (N42.6591, E9.4504). Finally, the specimens of *Macrostomum pusillum* Ax 1951 derive from a long-term outbred laboratory culture initially collected in April 2006 from the Laguna di Marano side in Lignano Sabbiadoro, Italy (N45.6916, E13.1311; note that the name *M. pusillum* is also taxonomically problematic, as outlined in [15]). Sampling in the San Rossore Regional Park was performed under permit 3299/7–2-1 of the Tenuta di San Rossore, Italy. The two other sites did not include national parks or other protected areas of land or sea. All species were kept in replicated populations in glass Petri dishes and fed with the diatom *Nitzschia curvilineata* Hustedt 1922. In every generation, 20 juvenile animals were added to a dish and allowed to grow for several weeks (four to five weeks for *M. spirale* and *M. hystrix* and three to four weeks for *M. pusillum*), after which again 20 juveniles were transferred to a new set of Petri dishes to start the next generation. Worms were kept in artificial seawater (ASW, Wiegandt) at 32‰ salinity for *M. spirale* and *M. pusillum*, and at 8‰ salinity for *M. hystrix*. All animals used for the transcriptomes were not older than two months.

### Experimental design

Since some genes may only be active during development or during regeneration, we wanted to obtain animals at various life-stages, so that a significant fraction of genes will be represented in the resulting transcriptomes. This also allowed us to identify genes that are DE between these life-stages and define candidate pools of genes relevant for specific functions (e.g. genes upregulated in adults vs. juveniles are good candidates for reproduction-related genes). We produced RNA samples for adults (A), hatchlings (H), and regenerants (R), using three biological replicates per condition and species, for a total of 27 RNA-Seq libraries (Fig. 2a). We defined adults as animals with clearly visible testes and collected 60 animals per replicate for *M. hystrix* and *M. spirale*, and 225 animals per replicate for *M. pusillum* (due to the smaller body size of this species, see Fig. 1). Hatchling samples consisted of a mixture of animals from various developmental stages, from freshly hatched flatworms up to early juvenile stages, but not having any visible gonads. We collected, on average about 330, 650, and 1100 hatchlings for each replicate of *M. hystrix*, *M. spirale*, and *M. pusillum*, respectively. Due to the large number of animals needed, hatchlings of *M. pusillum* and *M. spirale* were harvested at two time points, dissolved in Tri™ reagent (Sigma), and stored at − 80 °C until RNA isolation (see below). Animals used for the regenerant group were amputated at the level behind the ovaries (black dotted lines in Fig. 1) and then put in ASW with diatoms and allowed to regenerate for a variable amount of time before sampling to capture animals at various stages of regeneration. For *M. hystrix* and *M. spirale,* ten animals per replicate were amputated each day for six subsequent days, and on the seventh day, total RNA was isolated (6 × 10 = 60 animals per replicate). *M. pusillum* was treated in a similar way, but due to the smaller size and shorter regeneration time, five times 30 animals were amputated, and total RNA was isolated on the sixth day (5 × 30 = 150 animals per replicate).

### RNA isolation, library preparation and sequencing

Before extraction, worms were starved for 24 h to give them time to regurgitate consumed diatoms. Next, worms were gradually relaxed using a dilution series of 7.14% MgCl_2_ in water and then directly dissolved in Tri™ reagent (Sigma) by pipetting up and down. Subsequently, the extraction was performed as to the manufacturer’s recommendations, with the slight modification that we centrifuged the Tri™ reagent – Chloroform mixture for 20 min instead of the recommended 15 min. Quality checking, library preparation, and sequencing were performed by the Genomics Facility Basel at the Department of Biosystems Science and Engineering of the ETH Zürich in Basel. Libraries were prepared using the TruSeq® Stranded mRNA kit (Illumina) and sequenced as 101 bp paired-end reads on a HiSeq2500 sequencer (using the HiSeq® SBS Kit v4, Illumina).

### Transcriptome assembly

We used Rcorrector (commit 24940c9, [46]) with standard settings to correct for error due to inaccurate base calling, retaining reads that could not be corrected. Rcorrector is a k-mer-based correction technique that has been developed specifically for RNA-Seq data. It first constructs a De Bruijn graph of the reads and then assesses the coverage of the k-mers in the graph. K-mers with low coverage compared to other members of the path are likely due to sequencing error and are corrected [46]. After error correction, we trimmed sequencing adapters and low quality reads using Trimmomatic (version 0.36, command: 2:30:10:8:TRUE LEADING:5 TRAILING:5 SLIDINGWINDOW:4:15 AVGQUAL:30 MINLEN:36), removing low-quality regions and requiring an overall Phred score of 30. We then de novo assembled transcriptomes using Trinity (Version 2.6.6, [47]), with a k-mer size of 25, digital read normalisation, and with settings for stranded libraries.

### Removal of cross-contamination

All 27 libraries were multiplexed and ran on two lanes of the sequencer. To demultiplex reads, all libraries were assigned to their respective sample using dual combinatorial TruSeq kit indices (i.e. the 8 bp i5 and i7 indices D501-D508 and D707-D710, respectively). Our samples were only unique in one index with all i5 and two of the four i7 indices used to tag multiple species. It has recently become evident that this kind of indexing can lead to demultiplexing errors, due to so-called index hopping [48, 49]. During this process highly expressed reads from one library can cross-contaminate another library on the same lane and then appear to occur in that library at low counts. Because we used such indices and had a high sequencing coverage, this is likely a concern in our data. To mitigate the issue, we cleaned our assemblies using CroCo with default settings [50]. CroCo uses information about the levels of expression across assemblies to detect cross-contamination [50]. We removed transcripts that CroCo detected as contaminations and retained transcripts with an ambiguous signal to be conservative.

### Transcriptome quality assessment

To assess transcriptome quality, we ran TransRate (version 1.0.2, [33]), which maps the reads back to the assembly and calculates mapping metrics (e.g. if both read pairs map to the same transcript in the expected order), followed by BUSCO (version 2.0, [51]), which searches for the presence of a curated set of core conserved genes. Specifically, we ran the BUSCO analysis with the metazoan dataset consisting of 978 genes (version uploaded 2016-11-01). We determined the empirical insert size of our libraries by mapping the reads to the assemblies using bwa (version 0.7.17-r1188) and then extracting the mean insert size using Picard (version 2.20.2). We calculated the 21-mer distribution of the trimmed and corrected reads using jellyfish (option: -C, version 2.2.6) and recorded the number of distinct k-mers.

### Transcriptomes used for orthology detection and DE analysis

Since the employed orthology detection method operates on amino acid sequences (see next section), we first inferred open reading frames (ORFs) and their corresponding amino acid sequences, for the generated transcriptomes of *M. hystrix*, *M. spirale*, and *M. pusillum*. We used TransDecoder (version 2.0.1, [52]) in combination with Pfam searches (version 32.0) to retain transcripts with predicted proteins and kept only one ORF per transcript using the “--single_best_only” option. We then reduced the resulting predicted proteins using the CD-HIT clustering algorithm (version 4.7, [53]), set to cluster amino acid sequences with at least 99.5% identity and extracted the coding sequences corresponding to the clustered amino acids for DE analysis. These simplified transcriptomes were then used in the orthology detection pipeline and the DE analysis.

For *M. lignano,* we used a previously published genome-guided transcriptome assembly (Mlig_RNA_3_7_DV1.v3 [36]; with the method described in [12]) as a starting point for our analysis. Since many transcripts in *M. lignano* are trans-spliced [11, 12], the initial assembly had been modified to refine the gene models, predict open reading frames (ORF) using TransDecoder, and remove non-coding and repetitive regions by mapping to the reference genome (Mlig_RNA_3_7_DV1.v3.coregenes method described in [12]). In addition, Grudniewska et al. [36] provide a file containing the amino acid sequences for only the best ORF per transcript (generated using TransDecoder, Mlig_RNA_3_7_DV1.v3.genes.bestORF.pep). This data corresponds most closely to the amino acid data we generated from our de novo assemblies. We therefore also clustered this version of the assembly with CD-HIT at 99.5% sequence identity and again extracted the corresponding coding sequences.

We thus used four amino acid sequence datasets represented by the best ORF per transcript for our orthology detection and the corresponding coding sequence datasets for the quantification of expression and DE analyses. We refer to these datasets as the reduced transcriptome assemblies in the case of the three de novo assemblies and as Mlig_37v3 for the *M. lignano* assembly.

### Orthology detection

Our main aim with the orthology detection analysis was to identify homologous genes between the three species we sequenced for this study and the well-annotated transcriptome of *Macrostomum lignano*. We used OrthoFinder in amino acid mode (version 2.2.6, [41]), a method that infers whole sets of homologous transcripts (which we call orthogroups [OGs] throughout the text), based on a gene tree approach. We ran OrthoFinder with the “--msa” flag to use multiple sequence alignment instead of the default DendroBLAST. In this mode, OrthoFinder infers multiple sequence alignments for each cluster of putative homologs using MAFFT and then infers a gene tree using FastTree. To assess the influence of the clustering on the detected orthologs, we ran OrthoFinder with both the raw and the reduced transcriptomes as input (raw refers to the transcriptomes before CD-HIT clustering). We found that most transcripts were shared between the two approaches, with only a smaller fraction exclusive to one method (shared: 218,367, raw only: 20,119, CD-HIT only: 8768). For the following, we decided to use the CD-HIT clustered amino acid ortholog sets, since they are less complex and more amenable to downstream analyses.

### DE analysis

We conducted DE analysis between all three of our biological conditions, i.e. adults (A), hatchlings (H), and regenerants (R). However, of the three possible resulting contrasts, we here primarily focus on the comparison between adults vs. hatchlings (AvH), since this comparison permits to identify candidate transcripts that are DE in the context of reproduction. The comparisons between adults vs. regenerants (AvR) and regenerants vs. hatchlings (RvH) are mainly dealt with in the additional information, with the former comparison permitting to identify candidate transcripts that are DE during regeneration, while the latter comparison does not a priori represent a very informative contrast.

We quantified the expression of transcripts in our reduced transcriptomes since this allows for easier comparison of expression between species. Specifically, we mapped the trimmed and corrected reads used for transcriptome assembly (see above) to the coding sequences of the reduced transcriptome assemblies, using Salmon in quasi-mapping mode (version 0.9, [54]) and then inferred DE using DESeq2 (version 1.24.0, [55]). Filtering to remove genes with low expression can improve the power of DE analyses [55–58], and we took the following two-step approach, which uses the independent filtering feature of DESeq2. First, all of the data was run through the DESeq2 pipeline and all pairwise contrasts tested (AvH, AvR and RvH). The overall mean count thresholds identified by DESeq2 for each contrast were collected, and the minimum of these thresholds was then used to filter genes for multiple test correction within each contrast. This ensured that the same criteria were used in each analysis. The number of transcripts remaining after this filtering procedure is given in Table 1. Thus, only these remaining transcripts were tested for DE. Note that estimates of DE using de novo assembled transcriptomes can lead to biased estimates and should be interpreted cautiously [59].

### Detection of OGs with consistent DE signal between species

The inferred OGs can be combined with the DE analysis to identify gene families showing a consistent expression signal across all three species. We provide candidate gene sets from OGs with a consistent DE signal between adults and hatchling (Additional file 4: Table S1 and Additional file 8: Table S5 detailing the OGs and all annotation information for the OGs, respectively). These genes are not only conserved in sequence (as indicated by the fact that they are in the same OG) but also in aspects of their expression level, making them promising targets in the search for reproduction-related genes (if upregulated in adults) or genes essential for development (if upregulated in hatchlings). To be annotated, we required that an OG has at least one transcript per species that is DE in the contrast under investigation. We then categorised the OGs into those that showed a consistent signal between all DE genes versus those that showed conflicting signals.

### Reannotation of the *Macrostomum lignano* transcriptome

Previous studies have used RNA-Seq in *M. lignano* to identify groups of genes involved in reproduction [21–23] or neoblast function [6], but not all of these studies used the same reference transcriptome. We have therefore combined information from three selected RNA-Seq studies (see Fig. 2b-d) and transferred their annotations to the most up-to-date transcriptome (see above). Specifically, we included a study [21] that generated expression data for candidate genes primarily expressed in specific body regions (i.e. the testis, ovary, and tail region; referred to as the **positional dataset**), a study [6] identifying genes expressed in both neoblasts and germline tissue (referred to as the **neoblast dataset**), and a study [23] that compared expression between worms held in different social group sizes (isolated, pairs, and octets; referred to as the **social dataset**). We also identified transcripts that would be amplified using primers of existing ISH probes that had been designed based on previous transcriptome versions. In Additional file 13: “Reannotation of Mlig_37v3 transcriptome” we summarise the approaches used in these studies in some more detail and explain how we transferred these findings to the Mlig_37v3 transcriptome, to be subsequently transferred to our newly generated transcriptomes (see next section).

### Transcriptome annotation

We performed de novo annotation of the three transcriptomes using Trinotate (version 3.1.1, [60]), which performs a BLASTX search against a protein database (in our case Swiss-Prot and UniRef90 from the 2018_11 release) to assign an annotation. The tool also assesses the presence of signal peptides with SignalP (version 4.1, [61]), transmembrane domains with TMHMM (version 2.0, [62], and domain content with HMMER (version 3.1b2), against the Pfam protein family database (version 32.0, [63]). We also transferred annotations derived from the three RNA-Seq experiments, and the different ISH experiments performed with *M. lignano* (see the previous section), using our inferred OGs. For this, we assumed that genes sharing an OG have a similar function and transferred the annotations from the *M. lignano* genes to the other genes in the group. We allowed an OG to carry multiple annotations from within and across datasets.

### OG protein divergence by annotation

To investigate if certain OGs show more divergence at the sequence level, we estimated the protein divergence for each OG that contained all four species. We first filtered each OG alignment to only contain protein sequences that shared an aligned region (Additional file 17: Fig. S3A), leading to the removal of one OG that as a result contained only three species. For the remaining 11,330 OGs, we then calculated all pairwise protein distances using the *protdist* function of PHYLIP (version 3.697, [64]), using the JTT substitution model, and retaining only the between-species values (Additional file 17: Fig. S3B). To avoid choosing one representative sequence per species and OG, we summarised the protein distances of each species pair as the mean of all pairwise comparisons between their sequences (Additional file 17: Fig. S3C). Finally, we averaged the protein distances for all species pairs to obtain one estimate of protein divergence per OG. For further analysis, we excluded 16 OGs with a protein divergence of more than two substitutions per site, since these likely are close to saturation or represent misalignments. We then compared the distributions of protein divergences of annotated OGs (positional: testis [*n* = 726], ovary [*n* = 259] and tail region [*n* = 172]; neoblast: neoblast_FACS [*n* = 259] and neoblast-strict [*n* = 184]; social: OvI [*n* = 2215] and BOTH [*n* = 259]) against OGs without an annotation from each annotation source (positional [*n* = 10,151], neoblast [*n* = 10,159] and social [*n* = 8803]). To partially mitigate the pseudoreplication that might result from treating the OGs as independent samples we calculated the median protein divergence for all OGs without annotation and then performed a one-sample Wilcoxon signed-rank test against this value, additionally correcting for multiple comparisons using the Benjamini-Hochberg procedure [65].

### OG species composition by annotation

If reproduction-related genes evolve rapidly, then we expect few OGs with an *M. lignano*-derived annotation suggestive of sexual reproduction to be complete (i.e. contain all four species). In particular, we expect fewer OGs to contain *M. pusillum* since it is most distantly related to *M. lignano* (Fig. 1). In contrast, OGs with a neoblast-specific annotation are expected to be more conserved and we therefore expected them to be complete more often. To test this we compared the proportion of complete OGs of each annotation type to a random sample of all OGs. Additionally, we compared the proportion of OGs with a positional annotation containing only *M. lignano* and *M. spirale* to a random sample of all OGs. These OGs are candidates for genes responsible for the morphological differences between reciprocally and hypodermically mating species since the hypodermically mating *M. hystrix* is phylogenetically closer to *M. lignano* than to *M. spirale* (Fig. 1). Particularly these OGs could contain genes controlling the presence or length of the sperm bristles and the sperm brush, structures that are absent in hypodermically mating species (see drawings in Fig. 1).

We used a resampling approach to test whether OGs differed in their species composition based on their annotation. We sampled from all OGs that contained *M. lignano*, whether or not they were annotated. For each annotation tested, we then drew a random sample, equal to the number of OGs with the annotation, and recorded species in them (e.g. OGs containing all four species). We repeated this procedure 100,000 times and compared the resulting distribution to the empirical value. We used the proportion of draws where the empirical sample was smaller or larger than the draw as the *p*-value testing whether the annotated OGs have a higher or smaller number of genes with a particular species composition than expected. Note that transcripts can not be considered completely independent since i) they can be part of the same co-expression network and ii) linkage disequilibrium between them can constrain their evolution. Both of these effects can effectively lead to pseudoreplication, and the *p*-values should thus be interpreted with this caveat in mind. To correct for multiple testing, we used the Benjamini-Hochberg procedure and applied it to all p-values generated for each test within OGs set with a particular species composition. Resampling was done in R (version 3.5.0, CRAN). Visualisation of intersections between annotations was done using UpSet plots [66], as implemented in the R package UpSetR [67].

### Testing for enrichment in signal peptide

Since transcripts annotated as tail region specific in the positional dataset have previously been shown to contain a high number of seminal fluid proteins with evidence for secretion [40], we tested if OGs with a tail region annotation were enriched for a SignalP annotation. For this, we conducted a chi-square test, comparing the expected count of SignalP annotations (derived from the proportion of OGs with each annotation type) to the observed count. We then visually compared expected and observed counts visually to determine which annotation class was enriched.

### Phylogenetics

We used 1263 one-to-one orthologs identified by OrthoFinder (see above) to infer the phylogenetic relationship between our four species. We aligned the amino acid sequence of each ortholog using MAFFT (version 7.310 [68]) with the L-INS-i algorithm and concatenated the alignments with AMAS [69]. This resulted in an alignment of 615,314 amino acid sites with 13.6% missing data. We estimated a maximum likelihood phylogeny using IQ-TREE (version 1.5.5, [70]) with a separate partition for each gene and we inferred the best substitution model for each partition using ModelFinder [71] with the BIC criterion. We used ultrafast bootstrapping [72], combined with the Shimodaira–Hasegawa–like approximate likelihood ratio test [73] to assess support for bipartitions.

### Whole-mount ISH

We performed ISH for members of OG0014441, which contained i) the best blastn hit for the RNA815_7008 transcript of *M. lignano* [21] (Maclig_37v3@Mlig016310.g1, see Additional file 18: Table S11), ii) Machtx_20180703@G07456_i1, iii) Macspi_20180703@G161928_i1, and iv) Macpus_20180703@G35224_i1. For RNA815_7008, we added the T7 and SP6 sequences to the 5′ end of the published primers and we designed primers for the other species. PCR conditions were: 98 °C 30s; 35 x [98 °C 10s; 58 °C 30s; 72 °C 30s]; 72 °C 120 s, 4 °C 15 min. The resulting PCR products for *M. hystrix, M. spirale,* and *M. pusillum* were cloned into the pGEM-T vector (Promega, USA). Plasmids were extracted with the PureYield Plasmid Minipreps System (Promega, USA). PCR with the M13 primer set was performed on the plasmids to obtain the template DNA, which was cleaned up with the Wizard SV Gel and PCR Clean-Up System (Promega, USA) prior to probe synthesis. PCR fragments of *M. lignano* were purified with the Roche High Pure PCR Product Purification Kit (Sigma-Aldrich, USA). All four DNA templates were sequenced at Microsynth AG, Switzerland. The primer sequences and the sequenced data can be found in Additional file 19: Table S12. As a control for unspecific staining, we also performed all ISH using sense probes, which should not ligate to the target mRNA.

The ISH probes were synthesised with the Roche DIG RNA labelling kit (SP6/T7; Sigma-Aldrich, USA) using 6.5 μl of the template DNA in a 10 μl reaction. Probes were cleaned up with Micro Bio-Spin 6 Columns (Bio-Rad, USA), following the manufacturer’s protocol. Probes were diluted to 5 ng/μl in hybridisation mix and stored at − 80 °C. Whole-mount ISH was performed according to the WISH protocol described in [14] with the following modifications: i) animal relaxation with 7.14% MgCl_2_ × 6 H_2_O was prolonged to 22 min for *M. pusillum* and 70 min for *M. spirale*, both on ice*. M. hystrix* was relaxed in 0.1% Chlorethone (1,1,1-Trichloro-2-methyl-2-propanol) in 8‰ artificial seawater for 20 min on ice; ii) a decreasing methanol series instead of ethanol series was used; iii) protease treatment was shortened to 15 min for *M. hystrix* and *M. spirale* and to 10 min for *M. pusillum*. iv) The heat-fixation in PBSw was prolonged to 30 min (for *M. hystrix, M. spirale,* and *M. pusillum*). v) The temperature of the stringent Hybmix/SSC-buffer washing steps was increased to 64 °C for *M. hystrix, M. spirale,* and *M. pusillum.* These changes reduced background staining, which should facilitate additional investigations in the future.

## Supplementary information

**Additional file 1.** Amino acid alignment of one-to-one orthologs. Amino acid alignment used for the phylogenetic analysis. See Additional file 2 and 3 for the inferred phylogeny and for the IQ-TREE logfile, respectively.

**Additional file 2.** Maximum likelihood phylogeny. Phylogeny inferred using IQ-TREE. See Additional file 1 and 3 for the amino acid alignment used to infer this phylogeny and for the IQ-TREE logfile, respectively.

**Additional file 3.** IQ-TREE logfile. Logfile of the maximum likelihood analysis using the alignment in Additional file 1 and resulting in the phylogeny in Additional file 2.

**Additional file 4: Table S1.** Orthogroups (OG) inferred by Orthofinder.

**Additional file 5: Table S2.** DE results comparing adults versus hatchlings (AvH).

**Additional file 6: Table S3.** DE results comparing adults versus regenerants (AvR).

**Additional file 7: Table S4.** DE results comparing regenerants versus hatchlings (RvH).

**Additional file 8: Table S5.** OG annotations. Annotations for each OG are given. Annotation sources include the positional, neoblast, and social dataset as well as data from ISH probes and the DE analysis.

**Additional file 9: Figure S1.** Upset plot of the intersection of orthogroup (OG) annotations from the positional, neoblast, and social datasets. The dots and lines on the bottom right show which intersection is represented by the bar plots above it. The size of intersections is given above the bar plot. To the left of the intersection diagram, the absolute number of OGs per annotation is given.

**Additional file 10: Table S6.** Annotation of the Mlig_37v3 transcriptome. Annotations for each gene are given. Annotation sources include the positional, neoblast, and social dataset.

**Additional file 11: Table S7.** OG species composition by annotation.

**Additional file 12: Figure S2.** Sense probe control ISH. Unspecific staining in pharyngeal glands and in the tail regions. Transcripts RNA815_7008 (*M. lignano)*, Machtx_20180703@G07456_i1 (*M. hystrix*), Macspi_20180703@G161928_i1 (*M. spirale*), and Macpus_20180703@G35224_i1 (*M. pusillum*) of the orthogroup OG0014441 were used for sense in situ probe generation. Scale bars: 100 μm. Image taken by PB.

**Additional file 13:.** Reannotation of Mlig_37v3 transcriptome.

**Additional file 14: Table S8.** Results of DE reanalysis of the positional dataset.

**Additional file 15: Table S9.** Results of DE reanalysis of the social dataset OvI contrast.

**Additional file 16: Table S10.** Results of DE reanalysis of the social dataset OvP contrast.

**Additional file 17: Figure S3.** Representation of the method used to estimate protein divergence for each OG. Species are abbreviated as: lig = *M. lignano*, htx = *M. hystrix*, spi = *M. spirale* and pus = *M. pusillum*. A: Hypothetical protein alignment of an OG containing all four species. Protein divergence was calculated between all sequences that share an aligned region (indicated in black), thus excluding sequences that do not overlap (indicated with an asterisk, i.e. lig3*, spi2*). B: Matrix of all pairwise comparisons between the overlapping sequences in the OG, with letters denoting divergences between particular species pairs (e.g. a_1–4_ represent the protein divergence between the sequences of *M. lignano* and *M. hystrix*). C: Average protein divergences between species pairs in the OG (e.g. a is the average of all a-values in panel B). The divergence for the whole OG is then calculated as the average protein distance over all six species pairs.

**Additional file 18: Table S11.** A table of information for each primer pair used in the current study to transfer ISH expression pattern annotations to the Mlig_37v3 transcriptome assembly.

**Additional file 19: Table S12.** The ISH primers used in this study.

## Data Availability

The raw sequencing data generated for this study are available in the NCBI Sequence Read Archive repository with the following accession: PRJNA590781. The assembled transcriptomes, annotation files, and other supporting information are available in a zenodo repository at 10.5281/zenodo.3547572 The RNA-Seq datasets used for the reannotation of the *M. lignano* transcriptome are available under the following NCBI accessions: Positional:  PRJNA272894, Neoblast:  PRJNA330918, Social:  PRJNA511444.

## Abbreviations

AvH: Adults vs. hatchlings
AvR: Adults vs. regenerants
ASW: Artificial seawater
DE: Differentially expressed or differential expression
DIG: Digoxigenin
DNA: Deoxyribonucleic acid
FACS: Fluorescence-activated cell sorting
ISH: In situ hybridisation
mRNA: Messenger RNA
OGs: Orthogroups
ORFs: Open reading frames
OvI: Octets vs. isolated
OvP: Octets vs. pairs
PBS: Phosphate buffer saline
PCR: Polymerase chain reaction
RNA: Ribonucleic acid
RNA-Seq: Next-generation sequencing of RNA
RNAi: RNA interference
RvH: Regenerants vs. hatchlings
WISH: Whole-mount in situ hybridization
