# Supplementary figures and images for: RNA-Seq of three free-living flatworm species suggests rapid evolution of reproduction-related genes
Source: BMC Genomics. 2020 Jul 6;21:462. doi: 10.1186/s12864-020-06862-x (PMC7336406; doi:10.1186/s12864-020-06862-x)

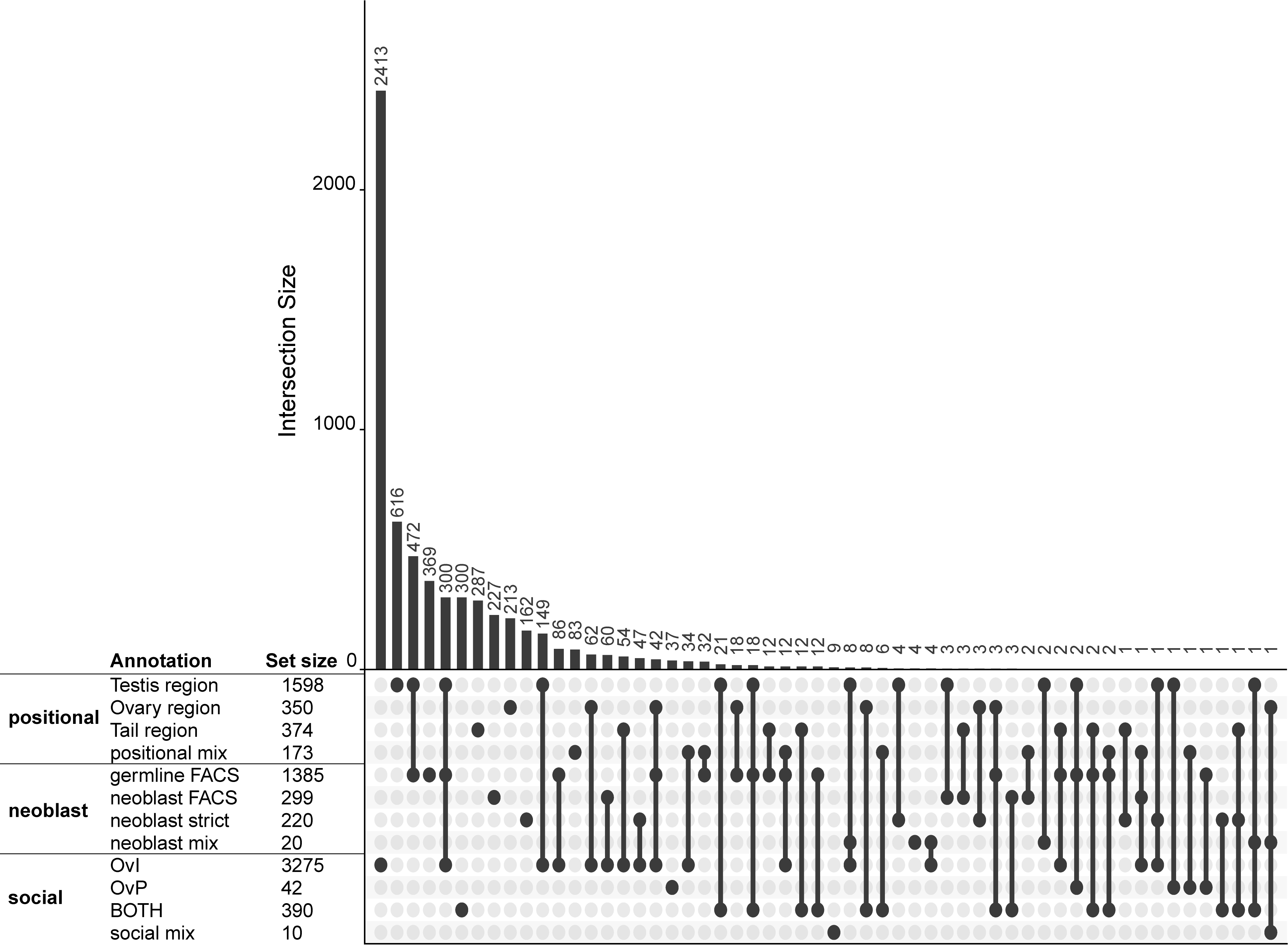

Supplement: Supplementary file 9 — Additional file 9: Figure S1. Upset plot of the intersection of orthogroup (OG) annotations from the positional, neoblast, and social datasets. The dots and lines on the bottom right show which intersection is represented by the bar plots above it. The size of intersections is given above the bar plot. To the left of the intersection diagram, the absolute number of OGs per annotation is given. [file 12864_2020_6862_MOESM9_ESM.png]

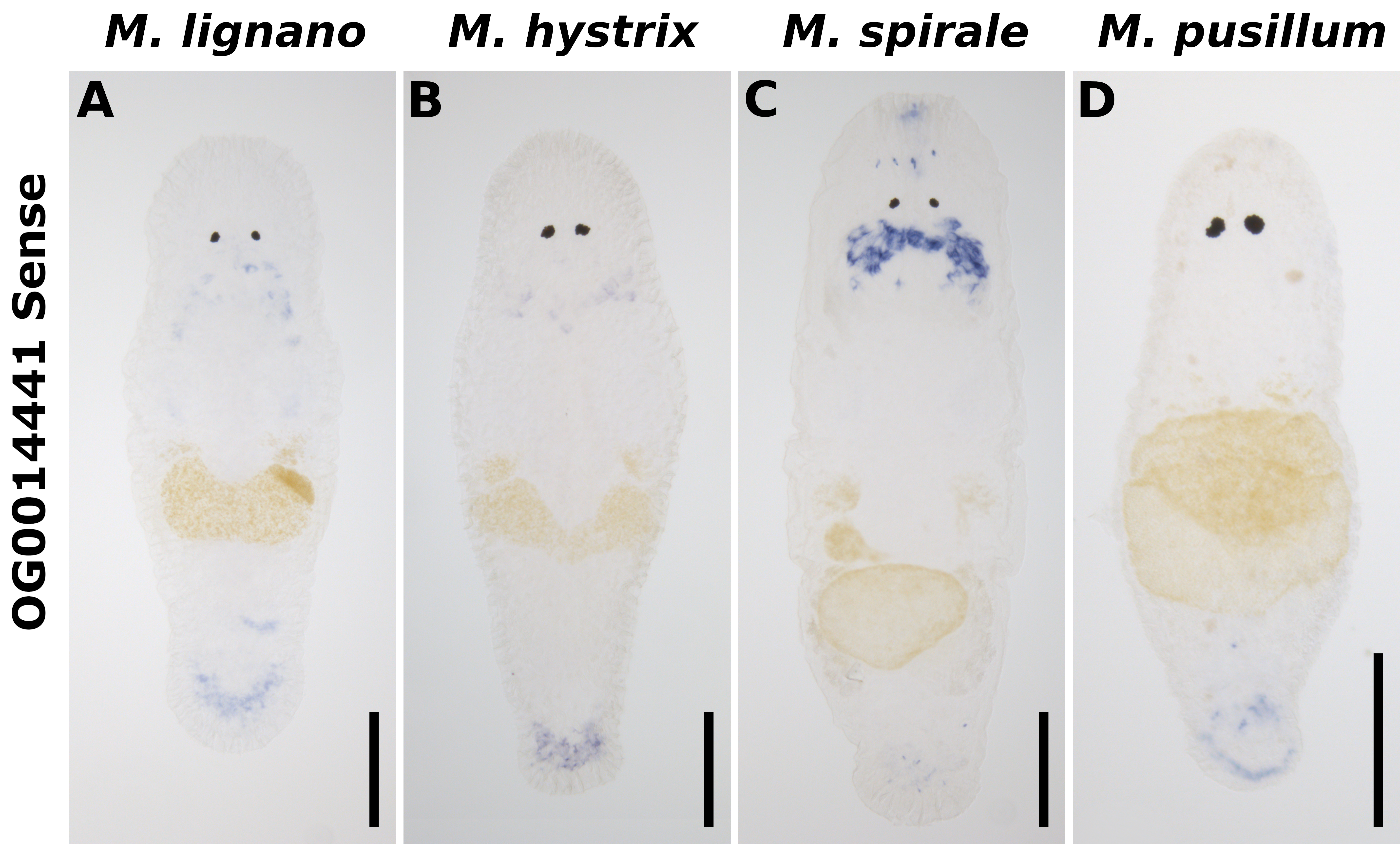

Supplement: Supplementary file 12 — Additional file 12: Figure S2. Sense probe control ISH. Unspecific staining in pharyngeal glands and in the tail regions. Transcripts RNA815_7008 (M. lignano), Machtx_20180703@G07456_i1 (M. hystrix), Macspi_20180703@G161928_i1 (M. spirale), and Macpus_20180703@G35224_i1 (M. pusillum) of the orthogroup OG0014441 were used for sense in situ probe generation. Scale bars: 100 μm. Image taken by PB. [file 12864_2020_6862_MOESM12_ESM.png]

**A**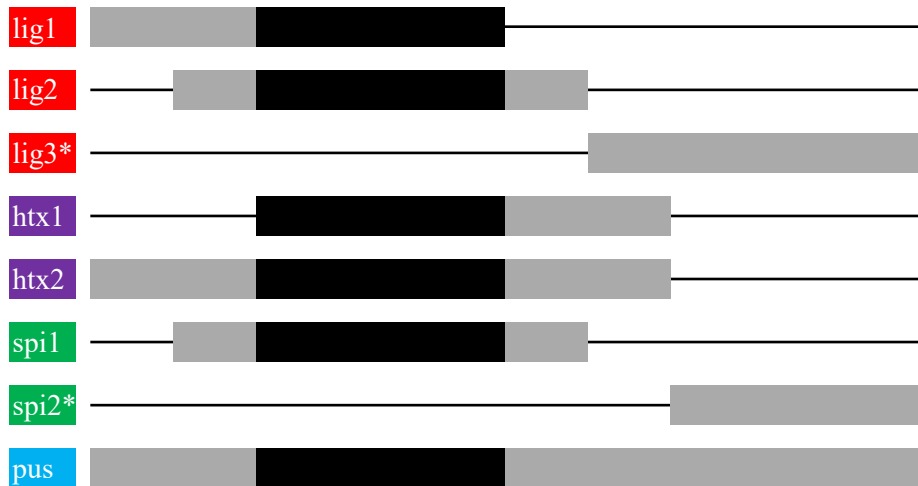**B**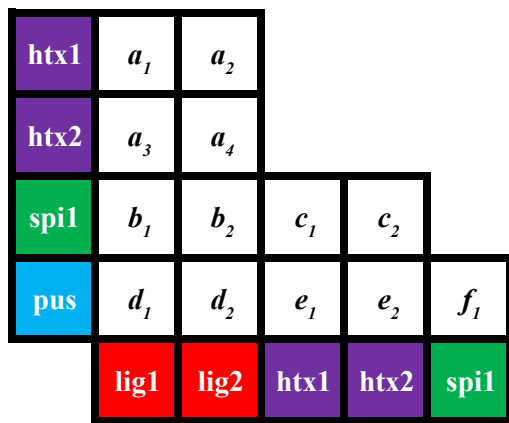**C**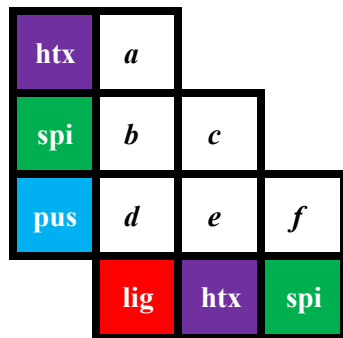

Supplement: Supplementary file 17 — Additional file 17: Figure S3. Representation of the method used to estimate protein divergence for each OG. Species are abbreviated as: lig = M. lignano, htx = M. hystrix, spi = M. spirale and pus = M. pusillum. A: Hypothetical protein alignment of an OG containing all four species. Protein divergence was calculated between all sequences that share an aligned region (indicated in black), thus excluding sequences that do not overlap (indicated with an asterisk, i.e. lig3*, spi2*). B: Matrix of all pairwise comparisons between the overlapping sequences in the OG, with letters denoting divergences between particular species pairs (e.g. a1–4 represent the protein divergence between the sequences of M. lignano and M. hystrix). C: Average protein divergences between species pairs in the OG (e.g. a is the average of all a-values in panel B). The divergence for the whole OG is then calculated as the average protein distance over all six species pairs. [file 12864_2020_6862_MOESM17_ESM.pdf]
