# Supplementary material for: RNA-Seq of three free-living flatworm species suggests rapid evolution of reproduction-related genes
Source: BMC Genomics. 2020 Jul 6;21:462. doi: 10.1186/s12864-020-06862-x (PMC7336406; doi:10.1186/s12864-020-06862-x)
Supplement: Supplementary file 13 — Additional file 13:. Reannotation of Mlig_37v3 transcriptome. [file 12864_2020_6862_MOESM13_ESM.pdf]

Additional file 13: Reannotation of Mlig_37v3 transcriptome

Introduction

In this document, we explain how we transferred the annotations that were made in *M. lignano* based on RNA-Seq and ISH using previous transcriptome versions of that species to the most up-to-date transcriptome version used in this study. Specifically, the MLRNA110815 transcriptome (available at http://www.macgenome.org/download/MLRNA110815; [1]), which has been used by Arbore et al. [2], Ramm et al. [3], and many follow-up ISH screens, has not previously been linked to the Mlig_RNA_3_7_DV1.v3 transcriptome assembly, so we have performed DE using these data using our reduced version of this assembly (Mlig_37v3) as a reference. In addition, we have transferred gene expression pattern annotations from ISH probes developed from older transcriptomes to transcripts of the Mlig_37v3 transcriptome. Finally, the transcripts of the transcriptome used in Grudniewska et al. ([4], assembly MLRNA150904) have been linked to the transcripts in the Mlig_RNA_3_7_DV1.v3 transcriptome using a genome-guided approach ([5]; with the method described in [6]). We were thus able to transfer the annotations of Grudniewska et al. [4] to the Mlig_37v3 transcriptome. We used the Mlig_37v3 transcriptome because it was also used for the orthology detection and estimating expression directly on these transcripts, thus makes comparisons across species easier. The detailed annotation of Mlig_37v3 can be found in Additional file 10 Tab. S6.

Positional dataset

The positional dataset of Arbore et al. [2] consists of four RNA-Seq libraries derived from animals cut into different fragments along the anterior-posterior body axis. The first sample was amputated anterior to the testes, the second sample anterior to the ovaries, the third sample was amputated posterior to the ovaries, and the fourth sample contained complete animals (fragments A-D, see main text Fig. 2). By comparing the expression in adjacent fragments (e.g. A vs. B), it is then possible to identify genes with higher expression in the body region contained only in the larger sample, thus allowing the identification of expression that is specific for the testis, ovary, and tail region.

DE analysis was performed with the same contrasts used in the original study [2]. Reads were trimmed using Trimmomatic, corrected using Rcorrector, expression quantified using Salmon

(version 0.9, [7]) in quasi-mapping mode with k-mer length 31, and then DE inferred using DESeq2 [8]. Due to the low quality of some 36bp single-end reads we employed a gentle trimming setting, a conservative minimum read length of 25, and an average quality score of 20 (2:30:10:8:TRUE LEADING:5 TRAILING:5 SLIDINGWINDOW:4:5 AVGQUAL:20 MINLEN:25). We calculated the expression level per body region as the percentage of reads mapping to a transcript to account for differences in sequencing depth among fragments, calculated identically to Arbore et al. 2015 as log_2(((reads mapping to transcript/total reads mapping within the segment) x 100) + 0.00001). We then assigned transcripts to the positional classes as defined in Arbore et al. [2].

In our reanalysis, we focused on genes with higher expression in the testis, ovary, and tail region but also annotated genes as non-DE or as "other" if the pattern did not match the previous classes. In Arbore et al. [2] two types of ovary region genes were defined, those that were DE only in the comparison of the B vs. C fragment and those that were additionally DE in the A vs. B fragment. We think that this latter category may have resulted from some ovary tissue having ended up in the second fragment, in spite of the fact that Arbore et al. [2] deliberately tried to cut those fragments somewhat anterior of the testis/ovary boundary, so as to avoid any ovary tissue being included. Moreover, this kind of pattern seems more likely for genes that are highly expressed in the ovary. Since both of these classes should yield ovary region candidates, we combined these two ovary classes into one class for our downstream analysis.

The reanalysis of the positional data resulted in a marked difference compared to the original results (Fig. A1A, Tab. A1). Arbore et al. [2] classified 93.8% of all transcripts as not DE, while our analysis only assigned 80.7% to that class. Most of this difference resulted from the fact that our analysis assigned 9.7% of all transcripts to classes referred to by Arbore et al. [2] as "other", while the value for their study was only 0.63%. We also assigned more transcripts to the testis, ovary, and tail regions, with the difference being most pronounced for one of the ovary classes and the tail region class (Tab. A1).

One source of disagreement appears to stem from the fact that transcripts with low expression are unreliably placed in the different classes. Indeed, when we excluded transcripts that had fewer than 50 reads mapped to them in the entire worm (called the D fragment in [2]), the differences between the two analysis were greatly reduced, with only 343 (0.59%) transcripts assigned to the "other" classes and the absolute numbers for the different positional classes becoming similar between [2] and our analyses (Fig. A2, Tab. A1).

Estimating expression of lowly expressed transcripts is notoriously difficult [9], and this problem is compounded by the lack of replication in Arbore et al. [2], which makes the use of statistical tools to call differential expression impossible. Differences between the analyses are likely also due to the fact that Salmon (used in this study) and RSEM (used in [2]), employ a different approach to calculate the effective length of transcripts when the transcript length is similar or smaller than the empirical fragment length (i.e. the insert size of the sequencing library). RSEM reduces the effective length of these transcripts to 1 and thus does not quantify their expression [10], while Salmon still quantifies such short transcripts [7]. To investigate this, we filtered our data to exclude all transcripts shorter than 300bp (with an empirical fragment length at 250bp on average), but this did not qualitatively change the observed differences between the analyses.

Finally, an important difference between the two analyses is that Arbore et al. used the options "--best --strata" when mapping the reads with bowtie. This option will only return the best alignment for each read and thus does not allow one to account for multi-mapping. Arbore et al. [2] correctly state that RSEM can account for multi-mapping in the analysis, but this is actually prevented by their bowtie settings. To verify this, we mapped all the reads in the D fragment to the Mlig_37v3 transcriptome and found that indeed all reads mapped uniquely (data not shown).

In conclusion we can say that even though there are considerable differences between the analyses, they are mainly affecting low-expression transcripts, which should be treated with caution anyway, especially because we are dealing with an unreplicated experiment. We retained all transcripts in our further analysis since it is easier for investigators to filter our results rather than repeating them. However, we caution against the use of candidates without prior inspection of their read counts. To facilitate this, we provide a column in the results table of the reanalysis grouping transcripts by count number (Additional File 14 Tab. S8).

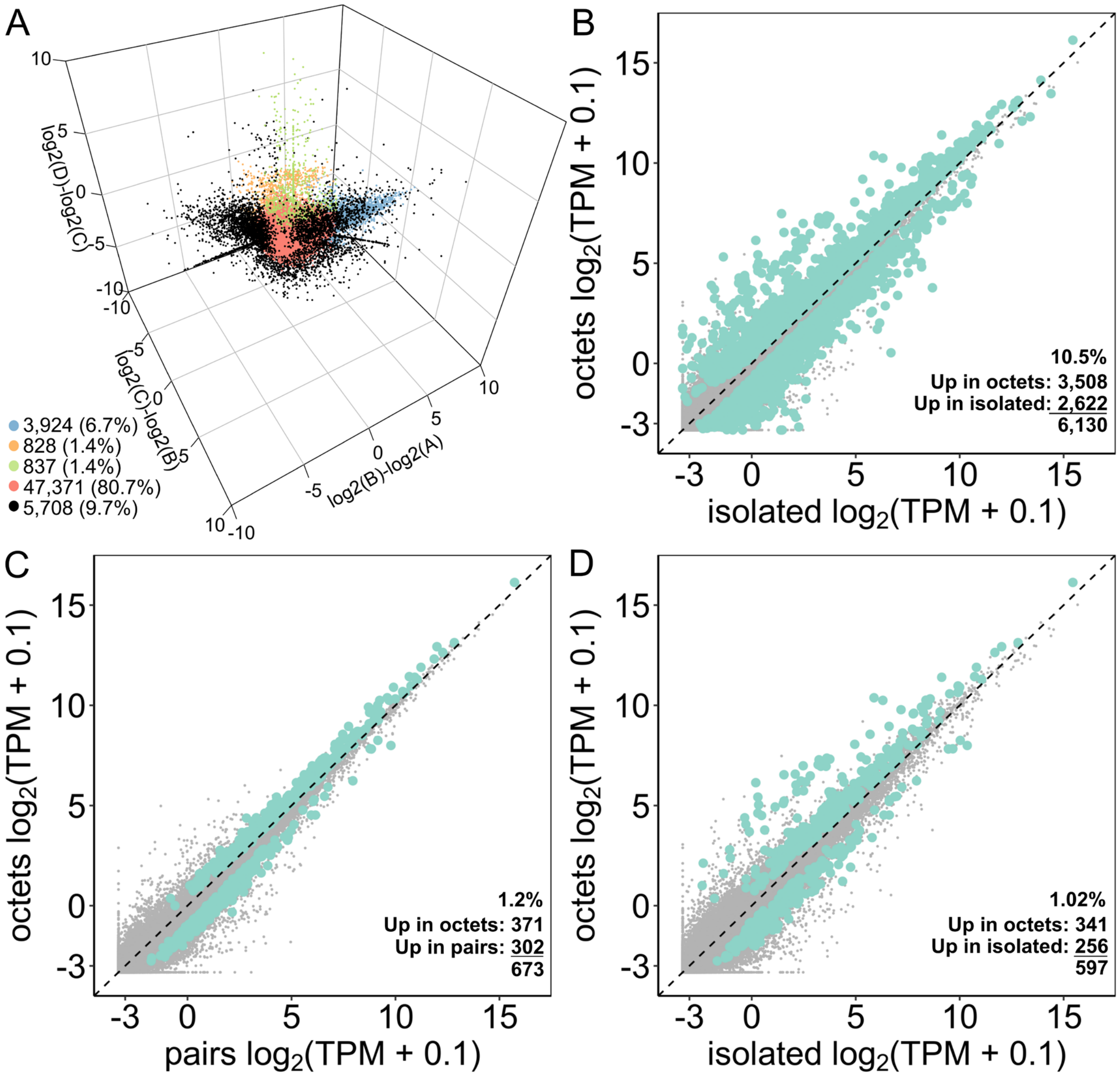

Fig. A1 Reanalysis of the positional and social RNA-Seq datasets. A: Differences in expression between different fragments for each gene. Each axis represents the *in silico* subtractions that were performed. Points are coloured by the positional classes to which the genes are assigned; blue – testis region, orange – ovary region, green – tail region, red – non-specific expression, black – other expression patterns. The inset numbers give the number and percentage of all genes belonging to each positional class. **B:** mean expression for transcripts in octets vs. isolated (OvI). **C:** mean expression for transcripts in octets vs. pairs (OvP). Coloured points in **B** and **C** show the genes with significantly different expression values. **D:** same points as in **B**, but coloured points show the transcripts that are differentially expressed both in OvI and OvP (BOTH). Inset values in the bottom right of panels **B**, **C**, and **D** give the number of genes that are more highly expressed in isolated, pairs or octets, as well as the total number of genes that were called as DE in each category. The values in the top left of **B**, **C**, and **D** give the percentages of DE genes out of the total (58,668).

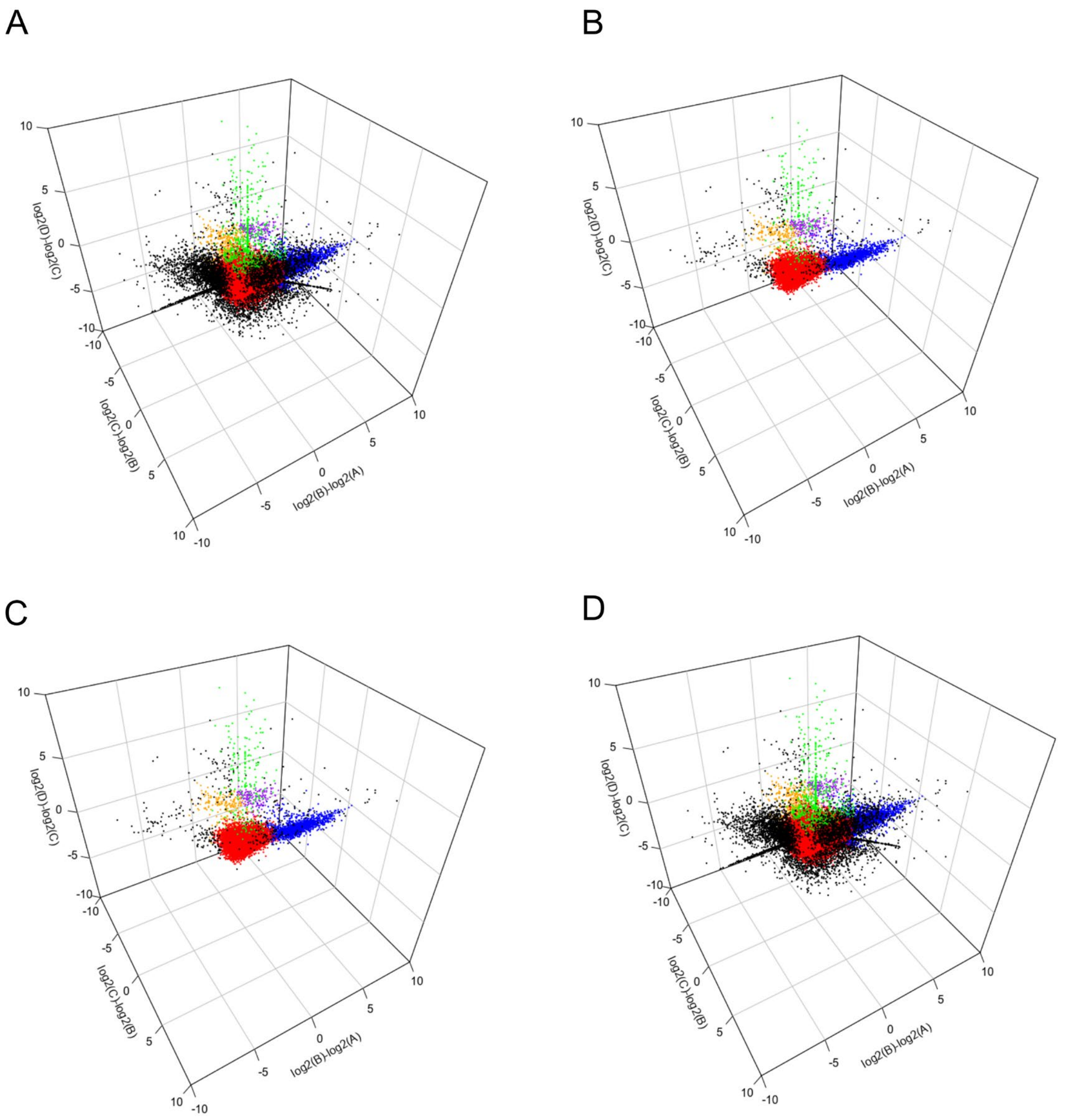

Fig. A2 Reanalysis of positional RNA-Seq dataset with different filtering options. Each plot shows the differences in expression between different fragments for each transcript. Each axis represents the *in silico* subtractions that were performed. **A:** All transcripts are shown. **B:** transcripts are subset to exclude those with low expression (<50 counts in the whole worm). **C:** transcripts are subset to exclude those with low expression and lengths <300bp. **D:** Transcripts are subset to exclude those with lengths < 300bp. See Table A1 for counts of genes in **A** and **B**.

Tab. A1 Counts and proportions of genes in each positional annotation class as defined in the original Arbore et al., 2015 paper. Counts in whole animal: Subset of data given by thresholds of the read counts in the "D fragment" (I.e. the whole worm) , meanBA: Mean difference in expression values between B and A fragments, meanCB: Mean difference in expression values between C and B fragments, meanDC: Mean difference in expression values between D and C fragments, Class: The code for the positional annotation category (as defined in Arbore et al., 2015), class_name: The name of the positional annotation category (as defined in Arbore et al., 2015), count: The count of the number of transcripts in this positional category, % of total: The percent of the total number of Mlig_37v3 transcripts (N = 58,668) that occur in the expression category, % of subset: The percent of the total within level of the "D_count_bin" column that occur in the expression category

Counts in whole animal	class	class_name	meanBA	meanCB	meanDC	count	% of total	% of subset	Arbore counts	Arbore % of total
[0 – max]	0,0,0	non_diff	-0.24	-0.12	0.02	47,371	80.74	80.74	70,064	93.78
	+,0,0	testes	4.10	-0.28	-0.35	3,924	6.69	6.69	3,360	4.50
	+,+,0	ovary2	3.11	3.82	0.43	187	0.32	0.32	127	0.17
	0,+,0	ovary1	-0.10	3.22	-0.04	641	1.09	1.09	323	0.43
	0,0,+	tail	-0.09	-0.41	3.33	837	1.43	1.43	366	0.49
	Other	other	-0.91	-0.25	-0.02	5,708	9.73	9.73	468	0.63
[0 – 49]	0,0,0	non_diff	-0.23	-0.19	-0.03	24,431	41.64	75.31		
	+,0,0	testes	3.29	-0.40	-0.34	1,559	2.66	4.81		
	+,+,0	ovary2	2.64	2.35	-0.87	13	0.02	0.04		
	0,+,0	ovary1	-0.33	2.82	-0.29	426	0.73	1.31		
	0,0,+	tail	-0.11	-0.52	2.70	645	1.10	1.99		
	Other	other	-0.92	-0.28	-0.19	5,365	9.14	16.54		
[50 – max]	0,0,0	non_diff	-0.24	-0.05	0.07	22,940	39.10	87.46		
	+,0,0	testes	4.64	-0.20	-0.36	2,365	4.03	9.02		
	+,+,0	ovary2	3.15	3.93	0.53	174	0.30	0.66		
	0,+,0	ovary1	0.37	4.01	0.46	215	0.37	0.82		
	0,0,+	tail	0.00	-0.05	5.43	192	0.33	0.73		
	Other	other	-0.77	0.24	2.61	343	0.58	1.31		

Neoblast dataset

Grudniewska et al. [4] used two strategies to identify transcripts upregulated in proliferating cells. First, they gamma-irradiated adult worms, thus killing all proliferating cells [11], and compared expression before and after irradiation to find genes with lower expression after irradiation. Second, they sorted cells stained for DNA from adults, hatchlings, and amputated heads using fluorescence-activated cell sorting (FACS), with gates designed to capture cells with a 2C and 4C DNA content. Cells with a 4C DNA content are late S-, G2-, and M-phase cells and thus proliferating. They compared expression between cells collected with the 2C and 4C gates to find genes upregulated in 4C cells. By then further comparing expression between 4C cells of adults against 4C cells of hatchlings and heads, they divided these candidates into

genes with biased expression in germline stem cells or neoblasts (somatic stem cells). Grudniewska et al. [4] thus, used three annotations: germline-specific genes identified using FACS sorting (germline_FACS) and two groups of neoblast specific genes (neoblast_FACS and neoblast-strict). The first neoblast group was also identified using FACS sorting only, but the second group was cross-referenced with irradiation results, and they concluded that these are the most stringent neoblast candidates.

Here we detail how we transferred the neoblast dataset annotations to the Mlig_37v3 transcriptome. Grudniewska et al. [4] performed DE analysis on transcripts clustered with Corset, which likely represent isoforms. For each transcript per Corset cluster, we first searched for the corresponding transcript(s) in the Mlig_RNA_3_7_DV1.v3.coregenes.bestORF.pep file (this corresponds to the transcriptome used in this study prior to CD-HIT clustering; see section "Transcriptomes used for orthology detection and DE analysis" in the main text) using the annotation provided by [5] and merged the annotation into the Mlig_37v3 transcript representing these hits. This was done to avoid losing annotations because an annotated Mlig_RNA_3_7_DV1.v3.coregenes.bestORF.pep transcript was clustered with an unannotated one by CD-HIT. When several transcripts were linked to one Mlig_37v3 transcript, we concatenated all annotations, potentially resulting in multiple annotations for the same transcript.

The majority of annotated transcripts could be assigned to an OG (Tab. A2). A small proportion of transcript clusters did not result in an annotation, either because we were not able to identify the transcripts in our Mlig_37v3 transcriptome or because multiple clusters were linked to the same Mlig_37v3 transcript (Tab. A2). But the majority of transcript clusters that did not result in an annotation did so because they were not assigned to an OG by Orthofinder (Tab. A2).

Since multiple transcripts could hit the same Mlig_37v3 transcript and then again, multiple Mlig_37v3 transcripts could be assigned to the same OGs, this resulted in some overlap between annotations. When an OG contained transcripts with a neoblast_FACS and transcripts with a neoblast-strict annotation, we annotated the OG as neoblast-strict. 20 OGs contained transcripts annotated as neoblast (neoblast_FACS and neoblast-strict) and also transcripts with the germline_FACS annotation. We annotated these OGs as neoblast_mix and excluded them from downstream analysis.

Tab. A2 Summary of the annotation transfer from the neoblast dataset. For the three types of annotations we transferred, we list the initial number of transcript clusters annotated by Grudniewska et al. (2016), followed by the total number of transcripts the clusters contained. We further list how many of these transcripts were represented in the Mlig_RNA_3_7_DV1.v3.coregenes assembly of Grudniewska et al. (2018) and how many transcripts in the Mlig_37v3 assembly were annotated based on this. The second to last row indicates how many transcripts in Mlig_37v3 were assigned to an OG with the percentage of transcripts in brackets. Finally, many transcripts were placed in the same OG and thus we give the total number of OGs annotated from the neoblast dataset. Note that if transcripts were annotated from multiple sources, we have counted them in each category with the exception of the final row, where these OGs were designated neoblast_mix as indicated by the last column.

No. of transcript clusters, transcripts or OGs	germline_FACS	neoblast_FACS	neoblast-strict	neoblast_mix
Initially annotated clusters	2,739	567	357	
Transcripts in the clusters	3,760	759	494	
Hit in Mlig_RNA_3_7_DV1.v3.coregenes	3,747	757	493	
Annotated transcripts in Mlig_37v3	3,429	716	405	
Assigned to an OG	2,650 (77.3%)	452 (63%)	371 (91.6%)	
Final OGs	1,385	299	220	20

Social dataset

The social dataset of Ramm et al. [3] consists of four treatments: isolated animals (I), animals that were isolated but then joined with a partner for 24h prior to RNA extraction (J), animals kept in pairs (P), and animals kept in octets (O). Each treatment consists of four biological replicates containing the RNA of 57 worms on average. In our reanalysis, we excluded the J treatment and compared expression between octets and isolated as well as octets and pairs.

The social dataset consists of high-quality, paired-end 100bp reads, which were trimmed using Trimmomatic, corrected using Rcorrector, expression quantified using Salmon (version 0.9, [7]) in quasi-mapping mode with k-mer length 31 and then DE inferred using DESeq2 [8]. Using these analyses, we could annotate transcripts as DE in octets vs. isolated (OvI), in octets vs. pairs (OvP), or in both comparisons (BOTH). We performed minimal pre-filtering to remove genes with no expression in any sample (i.e. a count of 0 in all samples), keeping ~97% of all transcripts for both the OvI and OvP comparisons. To correct for multiple testing, we set our false discovery rate (FDR) at a conservative 0.05. We used the independent filtering feature of DESeq2, which had not yet been implemented in the DESeq version used by Ramm et al. [3]. We also conducted the analysis with an FDR of 0.1, and without the independent filtering since this corresponds more closely to the analysis in Ramm et al. [3].

Overall, the proportion of DE transcripts for the OvI comparison are similar to the previously reported figures (i.e. 10.5%, compared to the 9.9% reported by Ramm et al. [3]; Figure 1B; Additional file 15: Tab. S9). Breaking the data down into patterns for each tissue region class, the absolute numbers of DE transcripts are also qualitatively similar to the previous values (Fig. A3, Tab. A3). We sought to determine whether the remaining differences could be explained by our analysis decisions. Specifically, we retrieved results for the OvI comparison using an FDR threshold of 0.1 and without applying the independent filtering in DESeq2. Although the values obtained with these settings are in some cases more similar to those reported by Ramm et al., they do not substantially differ, and we conclude that these decisions did not have a meaningful impact on the results (Tab. A3).

Fewer transcripts were found to be significantly DE in the OvP comparisons (Fig. A1C, Additional file 16: Tab. S10), and the overlap of the OvI and OvP datasets (i.e. BOTH) was quite substantial (Fig. 1AD). These results imply a relatively small number of transcripts that are exclusively DE in the OvP comparison (i.e. transcripts that respond in expression to an increase in social group size, rather than to the presence/absence of a mating partner). Since only a few transcripts were DE in OvP only, we focussed on transcripts in the OvI and the BOTH groups in our downstream analysis.

Similar to the neoblast dataset, we had some overlap between OG annotations because multiple Mlig_37v3 transcripts could be assigned to the same OG. When at least one transcript in an OG had a BOTH annotation, we annotated the OG as BOTH since this indicates that at least one transcript is sensitive to mating opportunity and the intensity of sperm competition. Ten OGs contained transcripts that were annotated OvI and additional transcripts annotated as OvP. It would be possible to annotate these OGs as BOTH but instead, we annotated them as social_mix to highlight that no single transcript within this OG had the BOTH annotation and excluded these OGs from downstream analysis.

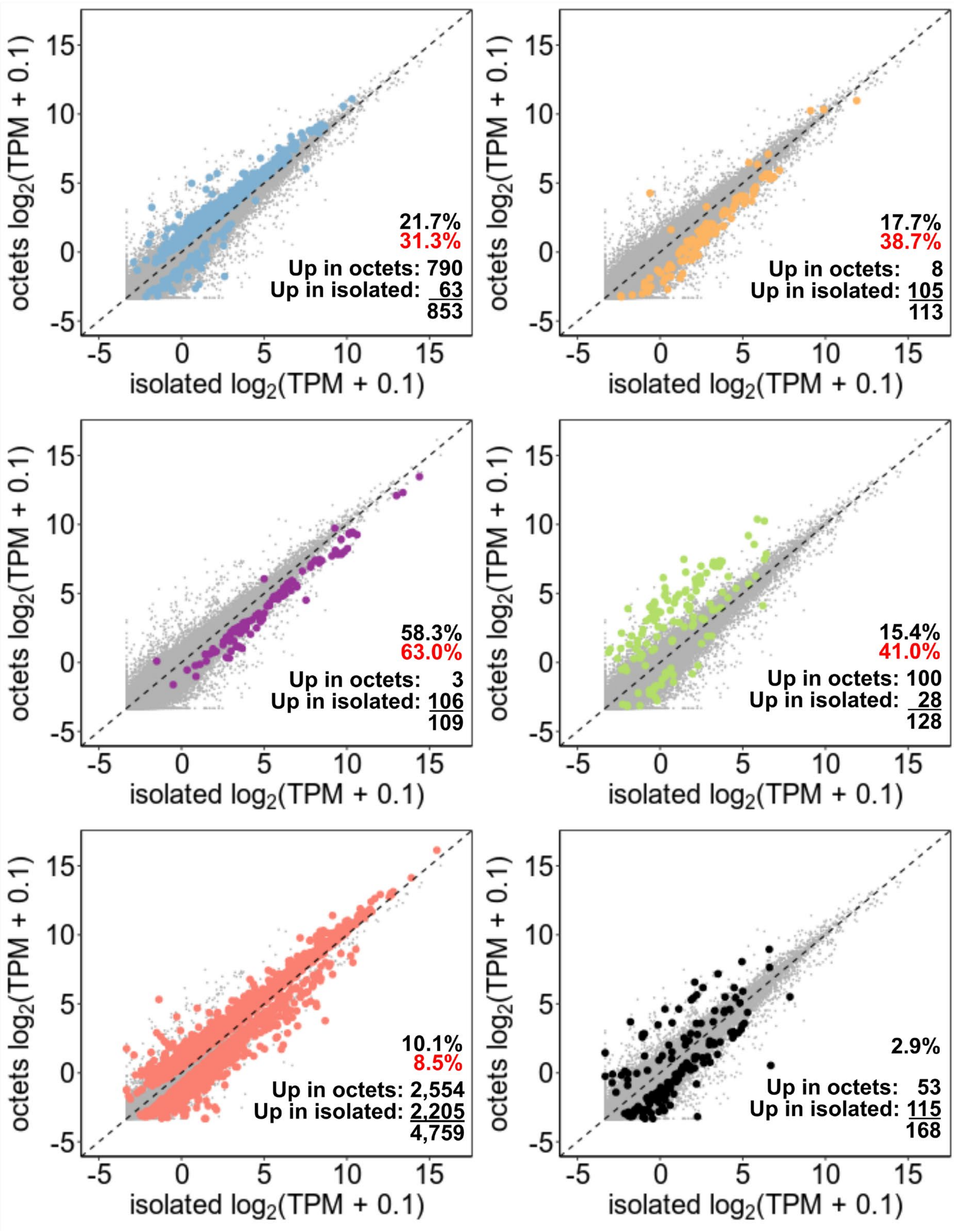

Fig. A3 Comparison of mean expression for transcripts in octets vs. isolated. Each panel shows genes called as DE in this comparison and also occur in the positional classes from the positional dataset. The colours are; blue – testis, orange – ovaries class 1, purple – ovaries class 2, green – tail, red – non-specific expression, black – other expression patterns. The inset values in the bottom right of each panel give the number of genes that are more highly expressed in octets and isolated worms, respectively, as well as the total number of genes that were called as DE from each positional class. The values in black in the top left of each panel give the percentage of genes from each positional class that were called as DE, in red are the percentages from the original study of [3] for comparison.

Tab. A3 Counts of DE genes from an analysis of worms kept in octets vs worms kept isolated (OvI). In order to compare results more closely to those of Ramm et al. [3], the analysis was also performed using an FDR threshold of 0.1 (as in Ramm et al., [3], the default of DESeq2) and without the independent filtering procedure of DESeq2. Given are the total number of tested transcripts in each group, the number and percentage that were either higher in octets or isolated, as well as the percentage of the total that were called as DE. We also give the breakdown of genes assigned to different tissue regions that are called as DE in the OvI comparison. Where possible, the corresponding overall proportions reported in Ramm et al. [3] are also reported in the final column.

**Category**	**Count**	**Higher in octets (%)**	**Higher in isolated (%)**	**% DE**	**% DE (from Ramm et al., 2019)**
Total	56,915	3,938 (6.9)	3,200 (5.6)	12.5	9.9
testes region	3,924	875 (22.3)	85 (2.2)	24.5	31.3
ovary 1 region	641	12 (1.9)	117 (18.3)	20.1	38.7
ovary 2 region	187	3 (1.6)	112 (59.9)	61.5	63.0
tail region	837	111 (13.3)	41 (5.9)	18.2	41.0
non-specific	47,371	2,861 (6.0)	2,673 (5.6)	11.6	8.5
Other	5,708	76 (1.3)	172 (3.0)	4.3	-

ISH data

Because most ISH studies have been conducted with the previous transcriptome assembly, we took a mapping approach to annotate the new *M. lignano* transcriptome using existing primer information. Primers used for designing ISH probes were collected from the literature [2, 3, 12, 13] and from personal correspondence with authors. Specifically, we collected information on i) the original assembly and transcript that was used to design the primers, ii) the estimated length of the PCR product, iii) the forward and reverse primer sequences, and iv) the ISH pattern observed. We focused on reproduction-related genes that showed expression in the gonads, prostate glands, and the antrum. In total 247 sets of primers representing 204 original transcripts were collected for which complete information was available (Additional file 18: Tab. S11). Many transcripts (92) had more than one set of primers. We mapped primer pairs to the reduced *M. lignano* transcriptome assembly (Mlig_37v3) with bowtie2 (version: 2.3.3.1; [14]). Maximum and minimum insert sizes were set as the original estimated product length +/- 100bp. No mismatches were allowed in the seed alignment. Mismatches may occur in the final alignment; three alignments had two mismatches, two alignments had indels with respect to the Mlig_37v3, and no alignments had more than two mismatches. The resulting .sam file

was parsed to identify primer pairs that mapped concordantly to only a single transcript. Because gene models are presumably improved in the newer transcriptome assembly and new isoforms may be identified, we treated each pair of primers as independent, even if they were designed from the same original transcript. Note that several primer sets have multiple annotations and the Mlig_37v3 transcripts inherited all annotations.

For those primer annotations that could not be transferred in the above way, we used blastn (version: 2.6.0; [15]) to identify the best hit in Mlig_37v3 of the original source transcript. We performed a one-way blastn with Mlig_37v3 as the database and MLRNA110815 as the query. We then ranked all hits first by e-value and used the bit score to break ties. For each MLRNA110815 transcript with ISH primer pairs that had not already been mapped to a Mlig_37v3 transcript, we identified the best blast hit and assigned the observed ISH pattern as an annotation to the Mlig_37v3 transcript.

Between 42.9 and 57.1% of primer pairs could be mapped to the Mlig_37v3 transcriptome (Tab. A4) and additional annotations could be transferred to Mlig_37v3 by blastn. The number of Mlig_37v3 transcripts that received an annotation was lower than the number of source transcripts. This is because i) different primer pairs from the same source transcript often mapped to the same Mlig_37v3 transcript, and ii) primer pairs from different source transcripts also often mapped to the same Mlig_37v3 transcript. For example, six separate primer pairs (RNA815_14562.a, RNA815_14562.b, RNA815_18395.a, RNA815_18395.b, RNA815_39625.b, and RNA815_64228.b) map concordantly to one Mlig_37v3 transcript (Mlig005822.g1, see also Additional file 18: Tab. S11). This Mlig_37v3 transcript was also the best blastn hit of another transcript (RNA815_42719), resulting in seven annotations of this single Mlig_37v3 transcript. The number of Mlig_37v3 transcripts that were finally assigned to each annotation category is given in Tab. A4. Note that some Mlig_37v3 transcripts will have multiple annotations and are represented multiple times, in total 147 Mlig_37v3 transcripts are annotated (Additional file 18: Tab. S11). The annotated transcripts occur in 77 orthogroups (Additional file 8: Tab. S5). The majority of orthogroups have representatives from all species except for those with antrum and prostate annotations (Tab. A4). While the sample size is quite small, this pattern is expected, since especially prostate transcripts are assumed to be under positive selection due to antagonistic coevolution.

Tab. A4 Summary of the annotation transfer from ISH patterns in *M. lignano*. Counts are given for each annotation category of how many primer pairs and transcripts there are, as well as how many could be transferred by primer mapping. Also given are the number of Mlig_37v3 transcripts and OGs that receive and annotation as well as how many of these OGs contain all species. Where relevant, the transcriptome assembly is noted in brackets. Note that some transcripts and orthogroups (OGs) are counted twice because they have multiple annotations.

**Annotation**	**Testes**	**Ovaries**	**Gonads**	**Antrum**	**Prostate**
No. primer pairs (MLRNA110815)	21	28	46	25	148
No. original transcripts (MLRNA110815)	20	27	43	22	110
No. (%) primer pairs that map	12 (57.1)	12 (42.9)	25 (54.3)	12 (54.5)	63 (43.6)
No. transcripts that receive an annotation from primer mapping (Mlig_37v3)	11	16	23	10	40
No. transcripts that receive an annotation from blastn (Mlig_37v3)	5	9	14	10	20
No. OGs that receive an annotation	14	19	24	11	16
No. (%) OGs that contain all species	9 (64.3)	14 (73.7)	14 (58.3)	5 (45.5)	7 (43.8)

References

1. Simanov D. Genomic resources for the flatworm model organism *Macrostomum lignano*. PhD dissertation. Utrecht University; 2014.

2. Arbore R, Sekii K, Beisel C, Ladurner P, Berezikov E, Schärer L. Positional RNA-Seq identifies candidate genes for phenotypic engineering of sexual traits. Front Zool. 2015;12:14.

3. Ramm SA, Lengerer B, Arbore R, Pjeta R, Wunderer J, Giannakara A, et al. Sex allocation plasticity on a transcriptome scale: Socially sensitive gene expression in a simultaneous hermaphrodite. Mol Ecol. 2019;00:1–21.

4. Grudniewska M, Mouton S, Simanov D, Beltman F, Grelling M, Mulder K de, et al. Transcriptional signatures of somatic neoblasts and germline cells in *Macrostomum lignano*. eLife. 2016;5:e20607.

5. Grudniewska M, Mouton S, Grelling M, Wolters AHG, Kuipers J, Giepmans BNG, et al. A novel flatworm-specific gene implicated in reproduction in *Macrostomum lignano*. Sci Rep. 2018;8:3192.

6. Wudarski J, Simanov D, Ustyantsev K, de Mulder K, Grelling M, Grudniewska M, et al. Efficient transgenesis and annotated genome sequence of the regenerative flatworm model *Macrostomum lignano*. Nat Commun. 2017;8:2120.

7. Patro R, Duggal G, Love MI, Irizarry RA, Kingsford C. Salmon: fast and bias-aware quantification of transcript expression using dual-phase inference. Nat Methods. 2017;14:417–9.

8. Love MI, Huber W, Anders S. Moderated estimation of fold change and dispersion for RNA-seq data with DESeq2. Genome Biol. 2014;15:550.

9. Zhang C, Zhang B, Lin L-L, Zhao S. Evaluation and comparison of computational tools for RNA-seq isoform quantification. BMC Genomics. 2017;18:583.

10. Li B, Dewey CN. RSEM: accurate transcript quantification from RNA-Seq data with or without a reference genome. BMC Bioinformatics. 2011;12:323.

11. De Mulder K, Kuales G, Pfister D, Egger B, Seppi T, Eichberger P, et al. Potential of *Macrostomum lignano* to recover from γ-ray irradiation. Cell Tissue Res. 2010;339:527–42.

12. Lengerer B, Wunderer J, Pjeta R, Carta G, Kao D, Aboobaker A, et al. Organ specific gene expression in the regenerating tail of *Macrostomum lignano*. Dev Biol. 2017;433:448–60.

13. Weber M, Wunderer J, Lengerer B, Pjeta R, Rodrigues M, Schärer L, et al. A targeted in situ hybridization screen identifies putative seminal fluid proteins in a simultaneously hermaphroditic flatworm. BMC Evol Biol. 2018;18:81.

14. Langmead B, Salzberg SL. Fast gapped-read alignment with Bowtie 2. Nat Methods. 2012;9:357–9.

15. Camacho C, Coulouris G, Avagyan V, Ma N, Papadopoulos J, Bealer K, et al. BLAST+: architecture and applications. BMC Bioinformatics. 2009;10:421.
